# Foodborne Illness Acquired in the United States—Major Pathogens, 2019

**DOI:** 10.3201/eid3104.240913

**Published:** 2025-04

**Authors:** Elaine J. Scallan Walter, Zhaohui Cui, Reese Tierney, Patricia M. Griffin, Robert M. Hoekstra, Daniel C. Payne, Erica B. Rose, Carey Devine, Angella Sandra Namwase, Sara A. Mirza, Anita K. Kambhampati, Anne Straily, Beau B. Bruce

**Affiliations:** Colorado School of Public Health, Aurora, Colorado, USA (E.J. Scallan Walter); Centers for Disease Control and Prevention, Atlanta, Georgia, USA (Z. Cui, R. Tierney, P.M. Griffin, R.M. Hoekstra, D.C. Payne, E.B. Rose, C. Devine, A.S. Namwase, S.A. Mirza, A.K. Kambhampati, A. Straily, B.B. Bruce); Cincinnati Children’s Hospital Medical Center, Cincinnati, Ohio, USA (D.C. Payne); University of Cincinnati College of Medicine, Cincinnati (D.C. Payne)

**Keywords:** foodborne illness, burden of illness, community incidence, foodborne disease, norovirus, *Salmonella*, *Campylobacter*, bacteria, enteric infections, food safety, viruses, United States

## Abstract

Estimating the number of illnesses caused by foodborne pathogens is critical for allocating resources and prioritizing interventions. We estimated the number of illnesses, hospitalizations, and deaths in the United States caused by 7 major foodborne pathogens by using surveillance data and other sources, adjusted for underreporting and underdiagnosis. *Campylobacter* spp., *Clostridium perfringens*, invasive *Listeria monocytogenes*, norovirus, nontyphoidal *Salmonella* serotypes, and Shiga toxin–producing *Escherichia coli* caused ≈9.9 million (90% credible interval [CrI] 5.9–15.4 million) domestically acquired foodborne illnesses in 2019. Together with *Toxoplasma gondii*, those pathogens caused 53,300 (90% CrI 35,700–74,500) hospitalizations and 931 (90% CrI 530‒1,460) deaths. Norovirus caused most illnesses (≈5.5 million illnesses, 22,400 hospitalizations), followed by *Campylobacter* spp. (1.9 million illnesses, 13,000 hospitalizations) and nontyphoidal *Salmonella* serotypes (1.3 million illnesses, 12,500 hospitalizations). *Salmonella* infection was the leading cause of death (n = 238). Foodborne illness estimates can inform policy and direct food safety interventions that reduce those illnesses.

Estimates of the number of illnesses caused by pathogens transmitted commonly through food (hereafter foodborne illnesses) provide valuable information for allocating resources and prioritizing interventions. Laboratory-confirmed illnesses are routinely reported to US public health departments, providing data that support trend assessments. However, those reports are limited because not all ill persons seek medical care or receive a diagnosis, and illnesses might not be reported to public health surveillance systems. To overcome reporting challenges, the Centers for Disease Control and Prevention (CDC) periodically estimates the number of foodborne illnesses by adjusting for undercounts in surveillance data (*1*–*3*). The estimates can be used to analyze health, economic, and other effects of foodborne illnesses.

Since the last estimates published in 2011 (*1*), new data and methods have become available, and new regulations and other interventions to prevent foodborne illness have been implemented. Moreover, culture-independent diagnostic tests (CIDTs) have been more widely adopted, increasing the likelihood of identifying pathogens (*4*).

The Interagency Food Safety Analytics Collaboration considers 4 pathogenic bacteria to be priorities for source attribution and actions by regulatory agencies: *Campylobacter* spp., *Listeria monocytogenes*, nontyphoidal *Salmonella*, and Shiga toxin–producing *Escherichia coli* (STEC) (*5*). In this assessment, we also included norovirus, *Clostridium perfringens*, and *Toxoplasma gondii* because all 7 pathogens were previously estimated to be the leading causes of domestically acquired foodborne illnesses, hospitalizations, and deaths in the United States (*1*,*6*). We provide regulators, policy makers, and others with updated estimates for 7 major foodborne pathogens as of 2019.

## Methods

We estimated the number of foodborne illnesses, hospitalizations, and deaths caused by *Campylobacter* spp., *C. perfringens*, invasive *L. monocytogenes*, norovirus, nontyphoidal *Salmonella*, and STEC and the number of hospitalizations and deaths caused by *T. gondii.* We produced separate estimates for STEC serogroup O157 and all other non-O157 STEC serogroups, for the top 5 *Salmonella* *enterica* serotypes (Enteritidis, I 4,[5],12:i:-, Javiana, Newport, and Typhimurium) and all other nontyphoidal *Salmonella* serotypes, and for non–pregnancy- and pregnancy-associated listeriosis. We did not estimate the numbers of *Toxoplasma*-induced illnesses because most infected persons were asymptomatic, and the number of persons experiencing mild illness was unknown.

### Illnesses

To estimate illnesses, we obtained counts of laboratory-confirmed illnesses and adjusted for undercounts caused by laboratory-confirmed illnesses not being reported to public health departments (underreporting) or caused by a lack of diagnosis because the ill person did not seek medical care or submit a specimen or because the laboratory did not test for or identify the causative agent (underdiagnosis). We made adjustments by using a series of pathogen-specific multipliers estimated by using specific methods and data sources (Appendix 1; Appendix 2).

To determine the number of illnesses caused by *Campylobacter* spp., nontyphoidal *Salmonella*, and STEC, we used 2017–2019 data from the Foodborne Diseases Active Surveillance Network (FoodNet, https://www.cdc.gov/foodnet). FoodNet conducts active, population-based surveillance of laboratory-diagnosed infections at 10 sites (≈15% of the US population). We estimated the number of laboratory-confirmed illnesses nationwide by applying the incidence obtained from FoodNet according to year and site to US population estimates. We randomly assigned *Salmonella* isolates with unidentified serotypes to 1 of 6 serotype groups according to weights equal to the proportions of isolates known to be in that group. Because of marked differences in clinical illness caused by O157 and non-O157 STEC serogroups, we imputed isolates with missing serogroup designations to O157 or non-O157 by using a supervised random forest model, which included patient demographics, symptoms, illness severity, travel information, and outbreak association (Appendix 1).

We used 2016–2019 data from the CDC’s nationwide *Listeria* Initiative surveillance system to determine the number of illnesses caused by invasive *L. monocytogenes*, defined as isolation of *Listeria* from a specimen collected from a normally sterile site (blood or cerebrospinal fluid) or product of conception (for pregnancy-associated illnesses: placenta, amniotic fluid, umbilical cord, or chorion). *L. monocytogenes* can also cause gastroenteritis and fever; however, fecal specimens are almost never tested for *Listeria* bacteria except occasionally during outbreaks. In surveillance systems, mother–infant pairs are counted as a single case; however, we counted both mother and infant as separate cases of illness if *L. monocytogenes* was isolated from that person’s specimen or if the mother or infant of a person with an invasive case had symptoms (e.g., fever, vomiting, or diarrhea). We proportionally distributed infants with unknown outcomes according to infants with known outcomes.

We used 10 years (2010–2019) of data from the Foodborne Disease Outbreak Surveillance System (FDOSS) to estimate the number of illnesses caused by *C. perfringens* because of high year-to-year variability in reported outbreaks. We derived incidence of norovirus illnesses by combining estimates from 2 studies of norovirus medical encounters; 1 study used active surveillance from Kaiser Permanente Northwest (2014–2016), and the other used administrative data from MarketScan Commercial and Medicare Supplemental databases (2001–2015) (*7*,*8*).

Because FoodNet conducts active surveillance, we assumed all laboratory-confirmed illnesses were reported. The CDC’s *Listeria* Initiative is a passive surveillance system, but we assumed all diagnosed cases were reported because of the severity of invasive listeriosis, an assumption supported by similar reported rates in FoodNet and non-FoodNet sites (*9*,*10*). For *C. perfringens*, we estimated the number of laboratory-confirmed illnesses by using an outbreak underreporting multiplier, which was the inverse of the proportion of laboratory-confirmed illnesses for pathogens ascertained through FoodNet also reported to FDOSS as outbreak-associated (Appendix 1). We did not adjust norovirus estimates for underreporting because of the source data.

To adjust for underdiagnosis resulting from variations in medical care seeking and specimen submission for *Campylobacter* spp., nontyphoidal *Salmonella*, and STEC infections, we created multipliers for each laboratory-confirmed case in surveillance according to characteristics (age group, sex, race, ethnicity, and presence of fever or bloody diarrhea) of persons with acute diarrheal illness who reported seeking medical care and submitting a fecal sample in the 2018–2019 FoodNet Population Survey (*11*). Acute diarrheal illness was defined as diarrhea (>3 loose stools in 24 hours) lasting >1 day or causing restricted daily activities, excluding persons who indicated their illness was caused by a long-lasting or chronic illness or condition (e.g., colitis, irritable bowel syndrome). For norovirus, we adjusted for underdiagnosis by estimating the percentage of FoodNet Population Survey respondents who had acute gastroenteritis (diarrhea with >3 loose stools in a 24-hour period beginning in the previous month) lasting <3 days who had sought medical care, similar to a previous study (*12*). We did not adjust for fecal sample submission because norovirus inputs were from persons seeking medical care who had submitted a fecal sample (*7*,*8*). We assumed persons with invasive *L. monocytogenes* infections had high rates of medical care seeking (90%) and specimen submission (100%).

We determined the percentage of laboratories that routinely tested onsite or offsite for specific pathogens by using data from FoodNet laboratory surveys (*13*). We estimated test sensitivity for *Campylobacter* spp., nontyphoidal *Salmonella*, and STEC separately for illnesses diagnosed by using cultures and CIDTs. Most CIDTs are PCR based and are highly sensitive (*14*); therefore, we assumed 100% CIDT sensitivity and estimated culture sensitivity by using data from FoodNet, which indicated the number of CIDT-positive tests that were also positive by reflex culture (cultures performed in response to a positive CIDT test). We used this number as the lower parameter of the program evaluation and review technique (PERT) distribution (Appendix 2). We did not adjust for specificity because we made estimates on the basis of persons who sought medical care; we considered the identified pathogen to be the most likely cause of illness. We assumed that most persons with listeriosis who submitted a specimen for testing would be tested for *L. monocytogenes*; we determined test sensitivity according to the sensitivity of the blood culture (*15*). For *C. perfringens*, we used the overall *Salmonella* underdiagnosis multiplier to adjust for underdiagnosis.

### Hospitalizations and Deaths

For *Campylobacte*r spp., nontyphoidal *Salmonella*, and STEC infections, we estimated the numbers of hospitalizations and deaths by using FoodNet surveillance data (2017–2019); we determined the percentage of illnesses causing hospitalization or death according to year and site and applied those percentages to the estimated number of laboratory-confirmed illnesses. We used the same approach for *L. monocytogenes* by using the *Listeria* Initiative (2016–2019) and *C. perfringens* by using FDOSS (2010–2019) data. We also estimated the number of pregnancy-associated infections causing fetal death (abortion, miscarriage, or stillbirth). We estimated rates of hospitalizations and deaths caused by *T. gondii* infections by using the Healthcare Cost and Utilization Project National Inpatient Sample (2016–2019) and those caused by norovirus by using both the National Inpatient Sample (hospitalizations) and the National Center for Health Statistics multiple-cause-of-mortality dataset (deaths) (*8*); we applied those data to the average US population during 2017–2019.

We adjusted all hospitalizations and deaths for underdiagnosis, except for those caused by norovirus. We adjusted *Campylobacter* spp., nontyphoidal *Salmonella*, and STEC underdiagnoses according to published studies reporting the percentage of hospitalized patients with nonspecific gastroenteritis diagnostic codes who submitted a fecal sample for bacterial culture, along with the frequency of laboratory testing and test sensitivity for that pathogen (*16*,*17*). We applied the *Salmonella* underdiagnosis multiplier for hospitalizations and deaths to estimate hospitalizations and deaths caused by *C. perfringens* and *T. gondii* infections. Because of the high hospitalization rate, we assumed the underdiagnosis multiplier for invasive *L. monocytogenes* hospitalizations and deaths was the same as that for illnesses.

### Domestically Acquired Foodborne Illnesses

For *Campylobacter* spp., *L. monocytogenes*, nontyphoidal *Salmonella*, and STEC infections, we used surveillance data from FoodNet and the *Listeria* Initiative to estimate the percentage of illnesses acquired while persons had traveled outside the United States. We considered the remaining percentages of illnesses to be domestically acquired. Because of the rapid onset and short duration of illness, we assumed that ≈100% of *C. perfringens* and norovirus illnesses were domestically acquired. We also assumed toxoplasmosis was mostly domestically acquired.

We estimated numbers of domestically acquired foodborne illnesses caused by *Campylobacter* spp., nontyphoidal *Salmonella* (including specific serotypes), STEC, *T. gondii*, or norovirus according to a structured expert judgment study (*18*). We assumed illnesses caused by *C. perfringens* to be 100% foodborne because illness counts were from foodborne outbreaks. We assumed *Listeria*-induced illnesses to be ≈100% foodborne.

### Uncertainty Analysis

We used empirical data, when available, to define entire distributions or parameters of distributions (Appendices 1, 2). When data were sparse, we made reasoned judgments according to context, plausibility, and previously published estimates. The parametric distribution used for most multipliers was a 4-parameter β (modified PERT) distribution. The first 3 parameters were low, modal, and high; the fourth parameter was related to the variability of the distribution. We fixed the last parameter at 4, which yields a simple PERT distribution. However, when describing the outbreak reporting multiplier, we used a value of 64, which produced a PERT distribution mean equal to the target mean of our multiplier distribution (Appendix 1). When combining distributions from 2 sources, we used random sampling with weights equal to the number of events from each source.

Uncertainty in the estimates is the cumulative effect of uncertainty of each of the model inputs. We iteratively generated sets of independent pathogen-specific adjustment factors and used those multipliers to estimate illnesses, hospitalizations, and deaths. Using 10,000 iterations, we obtained empirical distributions of counts corresponding to Bayesian posterior distributions and used those posterior distributions to generate a point estimate (posterior mean) and 90% credible intervals (CrIs). Because incidence of illnesses differed by location (e.g., FoodNet sites) and over time, we included those variables in the models, which led to larger CrIs than if we had assumed inputs represented independent random samples of a fixed US population. We used R version 4.1.1 (The R Project for Statistical Computing, https://www.r-project.org) for analyses. The code is available from the authors upon request.

## Results

Of 37.6 million estimated episodes of illness caused by *Campylobacter* spp., *C. perfringens*, invasive *L. monocytogenes*, norovirus, nontyphoidal *Salmonella*, and STEC infections, 9.9 million (90% CrI 5.9–15.4 million) illnesses were estimated to be acquired domestically and were foodborne (Table 1). Together with *T. gondii*, domestically acquired foodborne illnesses caused by those pathogens resulted in an estimated 53,300 (90% CrI 35,800–74,600) hospitalizations and 931 (90% CrI 530–1,460) deaths (Table 2).

**Table 1:** Estimated annual numbers of illnesses caused by major foodborne pathogens, United States, 2019*

Pathogen	Laboratory confirmed, no.†			% From international travel	% Foodborne	No. domestically acquired foodborne illnesses, mean (90% CrI)
		Multipliers‡				
		Underreporting	Underdiagnosis			
*Campylobacter* spp.	72,218§	1	52.8	19	57	1,870,000 (696,000‒3,760,000)
*Clostridium perfringens*	811¶	28.9	38.1	<1	100	889,000 (19,300‒3,090,000)
*Listeria monocytogenes#*						1,250 (1,080‒1,460)
NPA	656**	1	1.7	3	100	1,050 (911‒1,220)
PA, mothers	66**	1	1.7	3	100	106 (76‒134)
PA, infants	57**	1	1.7	3	99	92 (74‒122)
Norovirus	2,915,705††	1	10.1	1	19	5,540,000 (2,230,000‒10,300,000)
Nontyphoidal *Salmonella* spp. serotypes#				NA	NA	1,280,000 (866,000‒1,760,000)
Enteritidis	11,240§	1	38.8	22	80	297,000 (181,000‒453,000)
I 4,[5],12:i:-	3,721§	1	38.5	10	66	93,900 (46,500‒163,000)
Javiana	4,324§	1	39.2	5	56	91,800 (21,400‒215,000)
Newport	5,906§	1	38.4	7	74	179,000 (66,200‒463,000)
Typhimurium	6,287§	1	39.0	7	59	135,000 (63,000‒227,000)
Other	24,504§	1	39.6	11	50	484,000 (171,000‒853,000)
STEC#				NA	NA	357,000 (159,000‒648,000)
O157	5,069§	1	38.9	9	60	86,200 (31,400‒201,000)
Non-O157	15,648§	1	42.7	24	50	271,000 (97,200‒546,000)
All pathogens#	NA	NA	NA	NA	NA	9,940,000 (5,860,000‒15,400,000)

*Means and CrIs were rounded off; unrounded numbers are shown in Appendix 3 Table 1. CrI, credible interval; NA, not applicable; NPA, non–pregnancy-associated; PA, pregnancy-associated; STEC, Shiga toxin–producing *Escherichia coli*. †Estimates according to the average US population during 2017–2019. ‡Pathogen- and data source–specific multipliers used to adjust for underreporting and underdiagnosis. §Active surveillance data from Foodborne Diseases Active Surveillance Network (2017–2019), adjusted for geographic coverage. ¶Passive surveillance data on outbreak-associated illnesses from the Foodborne Disease Outbreak Surveillance System (2010–2019). #Total numbers of domestically acquired foodborne illness were the sum of the estimates for corresponding subgroups. **Passive surveillance data from the Centers for Disease Control and Prevention Listeria Initiative (2017–2019). ††Active surveillance from Kaiser Permanente Northwest (2014–2016) and administrative data from IBM MarketScan Commercial and Medicare Supplemental Databases (2001–2015) (*1**,**2*) applied to the US population during 2017–2019.

**Table 2:** Estimated annual numbers of hospitalizations and deaths caused by major foodborne pathogens, United States, 2019*

Pathogen	Hospitalizations				Deaths		
	% Total hospitalizations	Underdiagnosis multiplier	No. domestically acquired illnesses, mean (90% CrI)†		% Total deaths	Underdiagnosis multiplier	No. domestically acquired illnesses, mean (90% CrI)†
*Campylobacter* spp.	22.1‡	1.7	13,000 (4,730‒25,500)		0.3‡	1.7	197 (0‒585)
*Clostridium perfringens*	<1§	1.5	338 (0‒1,920)		0.1§	1.5	41 (0‒189)
*Listeria moncytogenes*¶			1,070 (922‒1,240)		NA	NA	172 (144‒204)
NPA	87.4#	1.7	920 (796‒1,070)		15.8#	1.7	166 (142‒195)
PA, mothers	70.4#	1.7	74 (58‒91)		0#	1.7	0
PA, infants	80.6#	1.7	74 (58‒103)		5.8#	1.7	6 (0‒10)
Norovirus**	<0.1	NA	22,400 (9,570‒39,900)		<0.001	NA	174 (76‒308)
Nontyphoidal *Salmonella* spp. serotypes¶			12,500 (8,250‒17,700)		NA	NA	238 (7‒602)
Enteritidis	27.7‡	1.5	2,970 (1,530‒4,980)		0.6‡	1.5	63 (0‒242)
I 4,[5],12:i-	28.7‡	1.5	957 (339‒1,770)		0.7‡	1.5	26 (0‒158)
Javiana	25.2‡	1.5	882 (79‒2,620)		0.1‡	1.5	5 (0‒30)
Newport	28.0‡	1.5	1,720 (507‒4,000)		0.3‡	1.5	17 (0‒116)
Typhimurium	29.3‡	1.5	1,520 (576‒2,700)		0.8‡	1.5	40 (0‒19)
Other	27.1‡	1.5	4,500 (1,550‒8,180)		0.5‡	1.5	88 (0‒309)
STEC¶			3,150 (1,440‒5,870)		NA	NA	66 (0‒217)
O157	43.1‡	1.5	1,730 (574‒4,070)		1.0‡	1.5	40 (0‒180)
Non-O157	16.2‡	1.5	1,410 (465‒3,020)		0.3‡	1.5	25 (0‒94)
*Toxoplasma gondii*††	<0.001	1.5	848 (173‒1,700)		<0.001	1.5	44 (9‒90)
All pathogens¶	NA	NA	53,300 (35,800‒74,600)		NA	NA	931 (530‒1,460)

*Means and CrIs were rounded off; unrounded numbers are shown in Appendix 3 Table 2 (https://wwwnc.cdc.gov/EID/article/31/4/24-0913-App1.pdf). CrI, credible interval; NA, not applicable; NPA, non–pregnancy-associated; PA, pregnancy-associated; STEC, Shiga toxin–producing *Escherichia coli*. †Estimates according to the average US population during 2017–2019. ‡Percentage of patients who were hospitalized or died; data are from the Foodborne Diseases Active Surveillance Network (2017–2019). §Percentage of patients who were hospitalized or died; data are from the Foodborne Disease Outbreak Surveillance System (2010–2019). ¶Total numbers of hospitalizations and deaths were the sum of the estimates for corresponding subgroups. #Percentage hospitalized and died from the Centers for Disease Control and Prevention Listeria Initiative (2017–2019). We estimated an additional 38 (90% CrI 28‒47) fetal deaths, of which 37 (90% CrI 27‒45) were estimated to be the results of domestically acquired foodborne illness. **Rates of hospitalizations and deaths from the National Inpatient Sample of the Healthcare Cost and Utilization Project (hospitalizations) and the , Centers for Disease Control and Prevention National Center for Health Statistics multiple-cause-of-mortality dataset (deaths) for norovirus (2) applied to the US population during 2017–2019. ††According to the rates of hospitalizations and deaths from the National Inpatient Sample (2016–2019) applied to the US population during 2017–2019.

### Foodborne Illnesses

Of the pathogens included in this assessment, norovirus was estimated to cause most (5.5 million/year) domestically acquired foodborne illnesses, followed by *Campylobacter* spp. (1.9 million/year), nontyphoidal *Salmonella* (1.3 million/year), *C. perfringens* (889,000/year), STEC (357,000/year), and invasive *L. monocytogenes* (1,250/year). Of the illnesses caused by *Salmonella*, 297,000 (23%) were estimated to be caused by serotype Enteritidis, followed by serotypes Newport (179,000 [14%]), Typhimurium (135,000 [11%]), I 4,[5],12:i:- (93,900 [7%]), and Javiana (91,800 [7%]). More STEC illnesses were caused by all non-O157 serogroups (271,000 [76%]) than by STEC O157 (86,200 [24%]). An estimated 1,050 non–pregnancy-associated and 198 (106 mothers, 92 infants) pregnancy-associated illnesses were caused by *L. monocytogenes*. For pathogens with available surveillance data, underdiagnosis multipliers ranged from 1.7 for invasive *L. monocytogenes* to ≈39 for nontyphoidal *Salmonella* and STEC O157, 42.7 for non-O157 STEC, and 52.8 for *Campylobacter* spp. (Table 1).

### Hospitalizations

Norovirus was the leading cause of hospitalizations resulting from domestically acquired foodborne illnesses (22,400/year), followed by *Campylobacter* spp. (13,000/year), nontyphoidal *Salmonella* (12,500/year), STEC (3,150/year), invasive *L. monocytogenes* (1,070/year), *T. gondii* (848/year), and *C. perfringens* (338/year). The order of *Salmonella* serotypes causing most hospitalizations was the same as that for illnesses. Among domestically acquired foodborne illnesses, STEC O157 caused more (1,730 [55%]) hospitalizations than all non-O157 STEC serogroups (1,410 [45%]), although the overall number of hospitalizations caused by non-O157 STEC was slightly higher (Table 2). Invasive *L. monocytogenes* caused 920 non–pregnancy-associated and 148 (74 mothers, 74 infants) pregnancy-associated hospitalizations.

### Deaths

Domestically acquired, foodborne nontyphoidal *Salmonella* caused ≈238 deaths each year, followed by *Campylobacter* spp. (197/year), norovirus (174/year), invasive *L. monocytogenes* (172/year), STEC (66/year), *T. gondii* (44/year), and *C. perfringens* (41/year). Among the annual deaths caused by nontyphoidal *Salmonella*, 63 (26%) were caused by *Salmonella* serotype Enteritidis, followed by serotypes Typhimurium (40 [17%]), I 4,[5],12:i- (26 [11%]), Newport (17 [7%]), and Javiana (5 [2%]). We estimated 166 non–pregnancy-associated deaths and 6 pregnancy-associated deaths (all among infants) caused by *L. monocytogenes* (Table 2). In addition, we estimated 37 (90% CrI 27‒45) fetal deaths.

## Discussion

We estimated that foods contaminated with *Campylobacter* spp., *C. perfringens*, invasive *L. monocytogenes*, nontyphoidal *Salmonella*, STEC, and norovirus caused ≈9.9 million domestically acquired foodborne illnesses in the United States each year circa 2019. Domestically acquired foodborne illness caused by those 6 pathogen groups plus *T. gondii* resulted in ≈53,300 hospitalizations and 931 deaths. Similar to estimates circa 2006, norovirus was the leading cause of domestically acquired foodborne illness (*1*). Norovirus, *Campylobacter* spp., and nontyphoidal *Salmonella* caused most hospitalizations; nontyphoidal *Salmonella, Campylobacter* spp., norovirus, and invasive *L. monocytogenes* caused most deaths. Pathogen rankings were similar to previous estimates except that *Campylobacter* spp. ranked higher for all outcomes (higher than *Salmonella* for illnesses and higher than norovirus for deaths); norovirus ranked higher than *Campylobacter* spp. and nontyphoidal *Salmonella* for hospitalizations, and *T. gondii* ranked lowest for both hospitalizations and deaths (*1*). Global estimates have also indicated norovirus as the most frequent cause of foodborne illness (*19*,*20*), and similar studies in other countries have reported *Campylobacter* spp. and nontyphoidal *Salmonella* as leading causes of bacterial foodborne illness (*Campylobacter* spp. caused more illnesses) (*21*–*26*). Nontyphoidal *Salmonella*, *Campylobacter* spp., and invasive *L. monocytogenes* have generally been the leading causes of death worldwide, the rank order varying by country. The estimated rank of foodborne norovirus deaths also varies.

After Food and Drug Administration–approved culture-independent syndromic panels were introduced in 2013, most clinical laboratories adopted CIDTs to diagnose enteric pathogens—a dramatic shift from culture-based methods (*27*). The shift toward rapid multipathogen diagnostic assays correlates with increased detection of infections that might have otherwise gone undetected and unreported in US surveillance systems (*13*). This shift, along with advances in analytical methods, might have contributed to higher numbers of estimated domestically acquired foodborne illnesses in this study compared with estimates published in 2011 (*1*): *Campylobacter* spp., 845,000 (90% CrI 337,000–1,610,000) in 2011 compared with 1,870,000 (90% CrI 696,000‒3,760,000) in this report; nontyphoidal *Salmonella*, 1,030,000 (90% CrI 645,000–1,680,000) compared with 1,280,000 (90% CrI 866,000‒1,760,000); STEC O157, 63,200 (90% CrI 17,600–150,000) compared with 86,200 (90% CrI 31,400‒201,000); and non-O157 STEC serogroups, 113,000 (90% CrI 11,500–287,000) compared with 271,000 (90% CrI 97,200‒546,000). Diagnosing infections caused by non-O157 STEC serogroups was especially challenging before the introduction of CIDTs. Although serogroup O157 caused most STEC hospitalizations and deaths resulting from domestically acquired foodborne illness, non-O157 serogroups caused 3-fold more illnesses that O157 and caused a substantial number of hospitalizations and deaths.

Advances in our approach to adjusting for underdiagnosis also contributed to higher numbers of estimated illnesses caused by *Campylobacter* spp., nontyphoidal *Salmonella*, and STEC. Previously, we used bloody diarrhea as a marker of severity and adjusted for underdiagnosis caused by medical care seeking and fecal sample submission behaviors separately for persons with bloody and nonbloody diarrhea (*1*). Although bloody diarrhea is associated with medical care seeking, other drivers include illness duration, recent foreign travel, age, and socioeconomic status (*28*–*30*). Therefore, we included additional factors in the current estimates. When compared with previous estimates (*1*), the magnitude of underdiagnosis multipliers increased in this report for *Campylobacter* spp. (30.3 vs. 52.8), nontyphoidal *Salmonella* (29.3 vs. ≈39), and STEC O157 (26.1 vs. 39), indicating that adjusting for bloody diarrhea alone might have previously underestimated diagnosis rates.

Estimates of the number of domestically acquired foodborne illnesses attributable to norovirus were similar to those previously described (*1*) (5,460,000 [90% CrI 3,230,000–8,310,000] in 2011 vs. 5,540,000 [90% CrI 2,230,000‒10,300,000] in this report) despite improved data sources and changes in methods. The number of illnesses caused by *C. perfringens* (966,000 [90% CrI 192,000–2,480,000] vs. 889,000 [90% CrI: 19,300‒3,090,000]) and invasive *L. monocytogenes* (1,590 [90% CrI: 557–3,160] vs. 1,250 [90% CI: 1,080‒1,460) were lower in this report compared with those described previously (*1*). Differences in the estimated number of *C. perfringens* illnesses were mostly caused by a lower number of outbreak-associated cases reported during the study period, whereas the lower number of estimated invasive *L. monocytogenes* illnesses was mostly caused by using a lower underdiagnosis multiplier (driven by higher estimates of specimen submission frequency). Compared with previous estimates (*1*), the lower numbers for hospitalizations (4,430 [90% CrI 2,630–6,670] vs. 848 [90% CrI 173‒1,700]) and deaths (327 [90% CrI 200–482] vs. 44 [90% CrI 9‒90]) caused by *T. gondii* in the current estimates mirror trends in hospital discharge data and serosurveys (*31*–*33*) and likely reflect the increased availability of antiretroviral drugs for HIV, which, along with other immunocompromising conditions, is a major risk factor for severe illness from *T. gondii* reactivation (*34*).

Estimates of the percentage of illness transmitted by food and the percentage acquired while traveling abroad can profoundly affect the results and have important limitations. Many foodborne pathogens can be transmitted by other modes; however, data used to estimate foodborne illness percentages are often lacking. We used data from a structured expert judgment (*18*). Although subject to limitations, including expert bias, this approach offered consistency among all pathogens and ensured that transmission by all possible modes added up to 100%. For most foodborne pathogens, percentages were lower in this report than previously estimated (*1*). For norovirus, the uncertainty interval was large, reflecting a lack of consensus among experts. We used surveillance data to estimate the percentage of travel-related illnesses for most pathogens; however, the most recent FoodNet Population Survey found international travel was associated with medical care seeking (*30*). Therefore, our use of surveillance data might overestimate travel-related illnesses. For hospitalizations and deaths from toxoplasmosis, we assumed that most cases were domestically acquired, but that possibility might be unlikely because birth outside the United States is a risk factor for *T. gondii* infections (*31*).

The first limitation of our study is that the underlying data came from a variety of sources, which had variable quality and representativeness. Only data on foodborne outbreak-related illnesses were available for *C. perfringens*; it is unknown if those data are representative of all illnesses. For *Campylobacter* spp., nontyphoidal *Salmonella*, and STEC, we used data from FoodNet, an active surveillance system representing a 15% convenience sample of the US population. However, the distribution of *Salmonella* serotypes is not geographically homogeneous. Estimates of norovirus and *T. gondii* relied on administrative data sources that are not nationally representative. Second, ≈10% of data inputs were from nonempirical data. When data were sparse, we made reasoned judgments according to context, plausibility, and previously published estimates (e.g., applying the multiplier for *Salmonella* to *C. perfringens* illnesses). Third, illnesses caused by some pathogens might be underrepresented (e.g., *T. gondii* or febrile gastroenteritis caused by *L. monocytogenes*). We estimated numbers of fetal deaths caused by *L. monocytogenes*; however, early spontaneous abortion or miscarriage might be underdiagnosed. Moreover, many illnesses caused by foodborne pathogens can result in sequelae. Fourth, the source data has many unquantified uncertainties. The FoodNet catchment area is a critical contributor of unquantified uncertainty because of geographic variation in incidence rates for all pathogens. The FoodNet Population Survey is also a source of uncertainty; the survey responses were likely subject to recall and other biases. Our estimates for medical care seeking and sample submission make assumptions about the representativeness of survey participants relative to the target populations. Furthermore, the definition of acute diarrheal illness among survey participants could be made more or less restrictive, leading to smaller or larger numbers of reported acute diarrheal episodes used to estimate medical care seeking and sample submissions, adding additional uncertainties.

In conclusion, we estimated the number of domestically acquired foodborne illnesses, hospitalizations, and deaths caused by 7 major pathogens in the United States as of 2019. Our estimates can be used as a platform to attribute illnesses to specific food categories and also to estimate societal and economic costs of foodborne illness and the numbers of persons who develop disease sequelae. When considered with other information, such as major food sources of illnesses, population groups affected, and costs of making foods safer, the estimates can be used to prioritize food safety interventions.

Appendix 1 Additional information for models and approaches used for foodborne illness acquired in the United States—major pathogens, 2019.

Appendix 2 Additional information for estimations and uncertainty model inputs used for foodborne illness acquired in the United States—major pathogens, 2019.

Appendix 3 Additional information for means and credible intervals used for foodborne illness acquired in the United States—major pathogens, 2019.
