# Correction: Vol. 31, No. 4

**DOI:** 10.3201/eid3204.C13204

**Published:** 2026-04

**Keywords:** errata, erratum, correction

Some of the summary data included in Appendix 2 was incorrect in Foodborne Illness Acquired in the United States—Major Pathogens, 2019 (Elaine J. Scallan Walter et al.). The article has been corrected online (https://wwwnc.cdc.gov/eid/article/31/4/24-0913_article).

