# Supplementary material for: Foodborne Illness Acquired in the United States—Major Pathogens, 2019
Source: Emerg Infect Dis. 2025 Apr;31(4):669–77. doi: 10.3201/eid3104.240913 (PMC11950263; doi:10.3201/eid3104.240913)
Supplement: Appendix 1 — Additional information for models and approaches used for foodborne illness acquired in the United States—major pathogens, 2019. [file 24-0913-Techapp-s1.pdf]

Article DOI: https://doi.org/10.3201/eid3104.240913
EID cannot ensure accessibility for supplementary materials supplied by authors. Readers who have difficulty accessing supplementary content should contact the authors for assistance.

Foodborne Illness Acquired in the United States—Major Pathogens, 2019

Appendix 1

Model Structures and Approach Used to Make Estimates

Background

This work updates estimates of the number of domestically acquired foodborne illnesses, hospitalizations, and deaths in the United States for select pathogens (*1*). Although some new approaches were implemented, the model structures remained similar; starting with the observed counts or incidence of illnesses, we accounted for biases by adjusting for factors, including underdiagnosis and underreporting, proportion of domestically acquired illness, and proportion of foodborne illness. Those factors were included in the models as probabilities and implemented as random draws from different distributions. In this appendix, we describe the model structures and the factors. Note that we use the term Bayesian in the broad sense. For example, we include approximate Bayesian computation (*2*), although we don’t make explicit use of that method.

Model Structures

Two broad types of models or approaches were used:

• **Surveillance data scaled-up approach:** This model scales counts of observed laboratory-confirmed illnesses up to an estimated number of illnesses, accounting for underdiagnosis and underreporting, factors that contribute to an illness not being diagnosed or reported to public health surveillance (Appendix 1 Figure 1). The surveillance scaled-up model was used to estimate illnesses, hospitalizations, and deaths for the pathogens listed (Appendix 1 Table 1).

• **Population data scaled-down approach:** This model scales populations at risk down to an estimated number of ill persons (Appendix 1 Figure 2). The scaled-down model was used

to estimate illnesses, hospitalizations, and deaths for norovirus and hospitalizations and deaths for *Toxoplasma gondii* (Appendix 1 Table 2).

Each model has subtypes that reflect the available data. The figures describe mathematical multipliers in the key subtypes are described and illustrated for the surveillance data scaled-up model (Appendix 1 Figures 3, 4, 5) and for the population scaled-down model (Appendix 1 Figure 6). All figures consist of a series of histograms that describe the distributions of simulated individual multiplicative factors as they are successively applied to elements of the burden estimates.

The following 4 distributions were used:

•**Degenerate distribution.** Degenerate distribution is a distribution of a degenerate random variable (i.e., one that has a single possible value). Degenerate distribution was used for population ratios and underreporting (when we thought there was no underreporting; i.e., underreporting multiplier = 1).

•**Empirical distribution.** An empirical distribution was simulated by using simple nonparametric bootstrapping, which is the random resampling of observed data with replacement. Empirical distribution was applied to survey or surveillance data.

•**Program Evaluation and Review Technique (PERT) distribution.** The PERT distribution, the location/scale family extension of the beta distribution, is very flexible and can be useful when limited information about a finite support distribution is available. It is defined by the upper and lower bounds, the most likely value (modal value or mode), and a fourth parameter that controls the flatness of the distribution (the default value is 4). The smaller the fourth parameter, the flatter the distribution. Equations are available to convert between the first 3 parameters. PERT distribution was used when data from survey or surveillance were not available or insufficient to account for uncertainty. Mean and confidence limits from literature were used as parameters for PERT distribution. When the mode and only 1 of the upper and lower bounds was available, we calculated the other 2 parameters according to the assumption that the odds of the upper was 1.5 and lower bounds 1–1.5 times the odds of the mode.

•**Posterior distribution.** Posterior distribution was generated from Bayesian regression models. Because we used distributions as model inputs, use of posterior distribution has the advantage of being approximate to the underlying true distribution.

In the model, successive factors are applied by multiplication to obtain proportional increases or decreases in the observed counts or incidence of illness. A random sample (n = 10,000) was drawn for a factor from a given distribution. Two distributions were multiplied sequentially depending on their positions in the model. Note that if a degenerate distribution was involved in the multiplication, a value must be applied to the corresponding value in other distributions. Take counts of illnesses from FoodNet as an example; the ratio of the average of the 2017–2019 US population to the 2017 Georgia population must be multiplied with the number of illnesses in Georgia in 2017. In addition, a Bayesian model predicted probability of medical care seeking for a patient must be multiplied with the count for that person. Domestically acquired foodborne burden estimates were described by using the mean and a quantile-based 90% confidence interval derived from the resulting distribution of the final product of all factors.

Factors in the Surveillance Data Scaled-Up Model

Observed Laboratory-Confirmed Illnesses for Estimates of Illnesses

Campylobacter, Shiga toxin-producing Escherichia coli (STEC) O157, non-O157 STEC, Salmonella nontyphoidal serotypes. We used 2017–2019 FoodNet data from 10 sites (Appendix 1 Table 3). A total of 30 year- and site-specific counts were used as the source distribution to produce 10,000 bootstrap draws. *Campylobacter* or nontyphoidal *Salmonella* illnesses in FoodNet were diagnosed by culture only, culture independent diagnostic tests (CIDTs) and reflex culture, and CIDT only. Becasue we considered CIDT as the standard in this work, the observed counts of illnesses for FoodNet pathogens are stratified as either culture-diagnosed or CIDT-diagnosed (only if CIDT test was positive) (Appendix 1 Figures 4, 5).

Seventeen percent of *Salmonella* nontyphoidal isolates had no information on serotypes. We randomly assigned those isolates to 6 serotypes with weights proportional to the 6 serotypes of all known nontyphoidal *Salmonella* spp.

For STEC, 4,152 (48%) isolates did not have information on the O antigen. Because STEC O157 and other non-O157 STEC strains have different symptom profiles, we developed a random forest model by using data from 4,494 isolates with a known O antigen to predict O157 vs non-O157 and by using patient demographics (age group, race, ethnicity, sex), symptoms (diarrhea, bloody diarrhea, fever), illness severity (hospitalization, hemolytic uremic syndrome), outbreak association, history of international travel, FoodNet site, and year of illness. This model was then applied to the 4,152 isolates to predict the *E. coli* serogroup.

Listeria monocytogenes. Observed laboratory-confirmed invasive *L. monocytogenes* infections were from the nationwide Centers for Disease Control and Prevention (CDC) *Listeria* Initiative surveillance system during 2016–2019. Invasive *Listeria* was defined as isolation of *L. monocytogenes* from a specimen collected from a normally sterile site (blood or cerebrospinal fluid) or product of conception (for pregnancy-associated illnesses: placenta, amniotic fluid, umbilical cord, or chorion). During surveillance, mother-infant pairs are counted as a single case; however, we counted both mother and infant as separate cases of illness if *L. monocytogenes* was isolated from a specimen from that person or if the mother or infant of an invasive case reported symptoms (e.g., fever, vomiting, diarrhea). Infants with an unknown outcome were proportionally distributed according to infants with a known outcome. Bootstrapping samples of the 4 annual counts were used in the analysis.

Clostridium perfringens. We used data from the 2010–2019 Foodborne Disease Outbreak Surveillance System (FDOSS). We used 10 annual counts of illnesses to produce 10,000 bootstrapping draws.

Year or Geography for Illnesses, Hospitalizations, and Deaths

The observed laboratory-confirmed illnesses were adjusted for surveillance year and FoodNet site population to obtain projected US laboratory-confirmed illnesses (Appendix 1 Table 3). Active surveillance is preferred over passive surveillance and outbreak surveillance in burden estimates. Using the 2017–2019 FoodNet data produced the estimated average illness burden over that period. We used the average of the 2017–2019 US Census population to account for variation caused by the year and geographic coverage of surveillances. For FoodNet data, observed laboratory-confirmed illnesses for a given year and a given site were multiplied by the ratio of the average 2017–2019 US Census population (326,763,427) to the population

size of that site in that year. For *L. monocytogenes* and *C. perfringens*, national data were used. Thus, only the year was accounted for by multiplying the observed laboratory-confirmed illnesses for a given year by the ratio of the average 2017–2019 US Census population to the US Census population in that year.

Underdiagnosis Multiplier for Illnesses

In laboratory-based surveillance, underdiagnosis can occur if the ill person does not seek medical care, a specimen is not submitted for laboratory testing, the laboratory does not test for the causative agent, or the test does not identify the pathogen. We created multipliers to account for underdiagnosis (medical care seeking and specimen submission, laboratory testing, and test sensitivity) (Appendix 1 Table 4). The specific approach used for each pathogen was data driven. The approach used for *Campylobacter* spp., STEC O157, non-O157 STEC, nontyphoidal *Salmonella* spp. serotypes and *L. mon0cytogenes* is described. Because of the lack of data, we assumed the underdiagnosis multipliers for *C. perfringens* were the same as that for nontyphoidal *Salmonella* serotypes.

Medical Care Seeking and Specimen Submission

***Campylobacter*, STEC O157, non-O157 STEC, *Salmonella* nontyphoidal serotypes.** The 2018–2019 FoodNet Population Survey collected demographics and symptoms among participants who had acute diarrhea illness, defined as diarrhea (3 loose stools in 24 hours) lasting >1 day or resulting in restricted activities, excluding respondents with a chronic illness in which diarrhea or vomiting was a major symptom and who said that their recent diarrhea or vomiting episode was caused by a chronic digestive issue (such as colitis or irritable bowel syndrome). The FoodNet Population Survey also asked each participant if they sought medical care and if they submitted a specimen, which allowed us to examine if demographic or symptom information could predict medical care seeking and specimen submission. We chose a Bayesian approach for this analysis because the posterior distribution matched our need to produce a distribution to inform a component of our model.

Using FoodNet Population Survey data, we developed Bayesian models for predicting probabilities of medical care seeking in all participants and another model for predicting probabilities of specimen submission among those who sought medical care. Both models had noninformative priors, 4 chains, and 5,000 iterations with a warm-up of 2,500 iterations. The

model for specimen submission included age groups, sex, race ethnicity, bloody diarrhea, fever, and the interaction term between bloody diarrhea and fever. The model for medical care seeking included 2 additional terms: the interaction between age groups and bloody diarrhea and the interaction between age groups and fever.

The two models were then applied to the 2017–2019 FoodNet surveillance data to predict the probabilities of medical care seeking and the probabilities of specimen submission for each patient. To reduce the burden of computing, 1 draw of the predicted probabilities was randomly made for each patient. The reciprocal of the predicted probabilities for a patient was then sequentially applied to the count of observed laboratory-confirmed illness of that person (i.e., $1 \times \frac{1}{P_{medical\ care\ seeking}} \times \frac{1}{P_{specimen\ submission}}$) (Appendix 1 Figure 1, panel A).

L. monocytogenes. We used the approach laid out previously to estimate the underdiagnosis multiplier (Appendix 1 Figure 3, panel B) (*1*). We assumed all *L. monocytogenes* cases were severe. Different from *Campylobacter* spp., STEC O157, non-O157 STEC, and *Salmonella* nontyphoidal serotypes, the multiplier for medical care seeking and specimen submission was calculated as the product of $\frac{1}{P_{medical\ care\ seeking}} \times \frac{1}{P_{specimen\ submission}}$

Laboratory Testing

***Campylobacter* spp., STEC O157, non-O157 STEC, *Salmonella* nontyphoidal serotypes.** The number of specimens receiving a laboratory test was derived from the FoodNet Laboratory Survey. Each site reported the proportion of isolates that were routinely tested by year and pathogen both on and off site. The proportion for the listed FoodNet pathogens was close to 1.

L. monocytogenes. Laboratory testing data for *L. monocytogenes* were lacking. We assumed that most persons with listeriosis who submitted a specimen were tested for *Listeria* according to disease severity.

Test Sensitivity

***Campylobacter* spp., STEC O157, non-O157 STEC, *Salmonella* nontyphoidal serotypes.** We reported CIDT-diagnosed illnesses. However, some *Campylobacter* spp. and *Salmonella* nontyphoidal isolates were tested by culture only. The culture-diagnosed illnesses needed to be converted to CIDT illnesses by accounting for sensitivity of culture against CIDT.

The pathogen-specific test sensitivities were estimated from the FoodNet surveillance data. CDC encourages laboratories to culture specimens with positive CIDT results (i.e., reflex culture or follow-up culture). In the 2017–2019 FoodNet surveillance data, ≈36% of *Campylobacter* spp. and *Salmonella* nontyphoidal isolates were tested by both CIDT and reflex culture, allowing us to estimate the sensitivity of reflex culture as the counts of culture-positive and CIDT-positive isolates divided by the sum of the counts of culture-positive and CIDT-positive isolates and counts of culture-negative and CIDT-negative isolates. Because the sensitivity of culture decreases with the time elapsed after specimen collection, reflex culture is considered to have lower sensitivity compared with culture at the time of specimen collection. Thus, we used the sensitivity of the reflex culture as the lower boundary of the PERT distribution for timely culture. The mode and upper boundaries were estimated on the basis of the assumption that the odds of the upper boundary was 1.5 and lower boundary was 1–1.5 times the odds of the mode.

Listeria monocytogenes. CDIT is not routinely performed for *L. monocytogenes*, and we did not identify any new publications describing test sensitivity for this bacterium. We used blood culture sensitivity from a published study that we cited in a previous report (*5*).

Underreporting Multiplier for Illnesses

***Campylobacter* spp., STEC O157, non-O157 STEC, *Salmonella* nontyphoidal serotypes.** FoodNet is an active surveillance system. Thus, we assumed that there was no underreporting for FoodNet pathogens.

L. monocytogenes. Considering the severity of *Listeria* infections, we assumed that there was no underreporting for *Listeria*-associated illnesses. This assumption is supported by the negligible difference in counts of illness from the *Listeria* Initiative within the FoodNet areas versus the counts from FoodNet.

C. perfringens. Of the CDC surveillance systems, only FDOSS includes illnesses associated with *C. perfringens*. We attempted to maximize the robustness of our approach because of the uncertainties around acquiring laboratory confirmation and determining outbreak association. To estimate the underreporting multiplier, we applied the methods developed as previously described (*1*). In brief, the FoodNet system has information on whether an illness is associated with an outbreak, which allowed us to calculate outbreak-associated laboratory-

confirmed illnesses. Underreporting multipliers can then be calculated as the ratios of total laboratory-confirmed illnesses to outbreak-associated laboratory-confirmed illnesses in FoodNet.

We used 5 years of FoodNet data (2015–2019) for 11 pathogens to ensure all pathogens had nonzero outbreak-associated illnesses. The ratios were calculated (Appendix 1 Table 5). The 2 extreme ratios from *Yersinia enterocolitica* (460.0:1) and *Cyclospora cayetanensis* (3.5:1) were dropped. We used a minimum (7:1), maximum (297:1), and median (25:1) of the 9 ratios to define the lower and upper boundaries and the mode. To specify a variance parameter, we computed medians of the ratios across states according to year and then the median of those medians across years. The medians analysis yielded a value of 28.88, which we used as the target mean of our multiplier distribution. With minimum, mode, and maximum values fixed, we looked for a variance parameter that forced a PERT mean of ≈28.88. The PERT variance parameter was chosen to equal 64, producing a PERT distribution with a mean of 28.86.

Proportion of Domestically Acquired Illnesses, Hospitalizations, and Deaths

***Campylobacter* spp., STEC O157, non-O157 STEC, *Salmonella* nontyphoidal serotypes.** Laboratory-confirmed illness related to international travel generally accounted for a small proportion of total laboratory-confirmed illness (Appendix 1 Table 6). FoodNet collected information on international travel history of patients. Because of the small number of patients with a history of international travel, the proportions of international travel according to year and state were not computed. Instead, we calculated the proportion of international travel in all FoodNet sites combined during 2017–2019 for each pathogen. This overall proportion was used as the modal value in the PERT distribution, and uncertainty was determined on the basis of a 50% increase or decrease from the modal value on an odds scale.

***L. monocytogenes*.** The *Listeria* Initiative recorded that 3.18% of patients traveled internationally. The difference between pregnant versus nonpregnant women was negligible.

C. perfringens. The proportion of persons with *C. perfringens* infections associated with international travel was determined. The proportion was assumed to be low because the incubation period for *C. perfringens* infection is short.

Proportion of Foodborne Illnesses, Hospitalizations, and Deaths

***Campylobacter* spp., STEC O157, non-O157 STEC, *Salmonella* nontyphoidal serotypes.** For the foodborne attribution proportion, a calibrated and synthesized distribution for

each pathogen was obtained from the results of an expert elicitation (*6*). In brief, 48 experts representing a wide range of professional and scientific backgrounds were assigned to pathogen panels according to their expertise, education, work history, professional interest, experience, and knowledge of the individual pathogens in the study. Experts provided 5th, 50th, and 95th percentile estimates for the proportion of domestically acquired illnesses that are transmitted through each of the 5 major pathways, including the foodborne pathway. Those estimates were used to produce point estimates and 95% uncertainty intervals for pathogens and pathways. The point estimates were used as modal values, and uncertainty limits were used as the low and high boundaries for PERT distribution in our analysis (Appendix 2).

L. monocytogenes* and *C. perfringens. Foodborne illness caused by *L. monocytogenes* was assumed to be high and an appropriate PERT distribution was applied. Foodborne illness caused by *C. perfringens* was assumed to be high (Appendix 1 Table 6).

Incidence Rates for Hospitalizations and Deaths

Incidence rates of hospitalizations were calculated by using surveillance data. The bootstrapping draws were according to those incidence rates. Incidence rates of hospitalizations and deaths among laboratory-confirmed illnesses caused by Campylobacter spp., STEC O157, non-O157 STEC, Salmonella nontyphoidal serotypes were calculated by year and site by using the 2017–2019 FoodNet data. Incidence rates of hospitalizations and deaths among patients with illnesses caused by *L. monocytogenes* were calculated by using 2016–2019 *Listeria* Initiative surveillance system data. Incidence rates of hospitalizations and deaths among patients with illnesses caused by *C. perfringens* were calculated by using 2010–2019 FDOSS data.

Underdiagnosis Multiplier for Hospitalizations and Deaths

As previously described (*1*), a subjective underdiagnosis multiplier of 2 was used to estimate hospitalizations and deaths. In this work, we improved our analysis to calculate an underdiagnosis multiplier by using available data.

An underdiagnosis multiplier was calculated for *Campylobacter* spp., STEC O157, non-O157 STEC, *Salmonella* nontyphoidal serotypes as follows. Using linked laboratory and hospital discharge data from a healthcare organization and its affiliated hospital in previous reports, 5,804 of 7,862 of adult patients and 351 of 882 child patients with a discharge code for infectious intestinal disease, nonspecific gastroenteritis, or both had a fecal sample submitted for bacterial

culture (*7*,*8*). Taken together, 6,155 (70%) of 8,744 patients submitted a specimen. Thus, 70% was used as the mode for the PERT distribution of the proportion of specimen submissions. The uncertainty was calculated on the assumption that the odds of the upper boundary was 1.5 and lower boundary was 1–1.5 times the odds of the mode. The distributions for laboratory test proportion and sensitivity were used for estimates of illnesses. The underdiagnosis multiplier for illnesses was used for hospitalizations and deaths caused by *L. monocytogenes*. The underdiagnosis multiplier for hospitalizations caused by FoodNet pathogens was used for *C. perfringens* because of limited data.

References

1. Scallan E, Hoekstra RM, Angulo FJ, Tauxe RV, Widdowson MA, Roy SL, et al. Foodborne illness acquired in the United States—major pathogens. Emerg Infect Dis. 2011;17:7–15. PubMed https://doi.org/10.3201/eid1701.P11101

2. Marin J-M, Pudlo P, Robert CP, Ryder RJ. Approximate Bayesian computational methods. Stat Comput. 2012;22:1167–80. https://doi.org/10.1007/s11222-011-9288-2

3. Burke RM, Mattison CP, Marsh Z, Shioda K, Donald J, Salas SB, et al. Norovirus and other viral causes of medically attended acute gastroenteritis across the age spectrum: results from the medically attended acute gastroenteritis study in the United States. Clin Infect Dis. 2021;73:e913–20. PubMed https://doi.org/10.1093/cid/ciab033

4. Burke RM, Mattison CP, Pindyck T, Dahl RM, Rudd J, Bi D, et al. Burden of norovirus in the United States, as estimated based on administrative data: updates for medically attended illness and mortality, 2001–2015. Clin Infect Dis. 2021;73:e1–8. PubMed https://doi.org/10.1093/cid/ciaa438

5. Mylonakis E, Hohmann EL, Calderwood SB. Central nervous system infection with *Listeria monocytogenes*. 33 years' experience at a general hospital and review of 776 episodes from the literature. Medicine (Baltimore). 1998;77:313–36. PubMed https://doi.org/10.1097/00005792-199809000-00002

6. Beshearse E, Bruce BB, Nane GF, Cooke RM, Aspinall W, Hald T, et al. Attribution of illnesses transmitted by food and water to comprehensive transmission pathways using structured expert judgment, United States. Emerg Infect Dis. 2021;27:182–95. PubMed https://doi.org/10.3201/eid2701.200316

7. Scallan E, Griffin PM, McLean HQ, Mahon BE. Hospitalisations due to bacterial gastroenteritis: a comparison of surveillance and hospital discharge data. Epidemiol Infect. 2018;146:954–60. PubMed https://doi.org/10.1017/S0950268818000882

8. Scallan Walter EJ, McLean HQ, Griffin PM. Hospital discharge data underascertain enteric bacterial infections among children. Foodborne Pathog Dis. 2020;17:530–2. PubMed https://doi.org/10.1089/fpd.2019.2773

Appendix 1 Table 1. Pathogens for which surveillance data were used to estimate illnesses, hospitalizations, and deaths by using the surveillance data scaled-up model

Foodborne Diseases Active Surveillance Network (FoodNet)	*Listeria* Initiative	Foodborne Disease Outbreak Surveillance System (FDOSS)
Campylobacter spp.<br>Shiga toxin-producing *Escherichia coli* (STEC)<br>STEC O157<br>non-O157 STEC<br>Nontyphoidal *Salmonella* spp. serotype<br>Enteritidis<br>I 4,[5],12:i:-<br>Javiana<br>Newport<br>Typhimurium<br>Other	Invasive *Listeria monocytogenes;* nonpregnancy-associated listeriosis; pregnancy-associated listeriosis (mothers and live-born infants); fetal deaths	*Clostridium perfringens*

Appendix 1 Table 2. Pathogens for which administrative and other data sources were used to estimate illnesses, hospitalizations, and deaths by using the population data scaled-down model

Published studies*	National Inpatient Sample†
Norovirus	*Toxoplasma gondii*

*Published studies used for norovirus in scaled-down model (*3*,*4*).
†National Inpatient Sample database developed by the Healthcare Cost and Utilization Project (https://hcup-us.ahrq.gov/nisoverview.jsp).

Appendix 1 Table 3. Data source (distribution) for counts of illnesses and year and geography of the surveillance data scaled-up model for 6 pathogens*

	Observed counts of illness		Year or geography
Pathogen	Test method	Data source, distribution	Data source, distribution
Campylobacter spp.	Culture, CIDT	FoodNet, by year and site (empirical)	Ratio of average 2017–2019 US population to FoodNet site population, by year (degenerate)
STEC O157	CIDT		
non-O157 STEC	CIDT		
Nontyphoidal Salmonella spp. serotypes	Culture, CIDT		
Listeria monocytogenes	Culture	Listeria Initiative, by year (empirical)	Ratio of average of 2017–2019 US population to US population, by year (degenerate)
Clostridium perfringens	Culture	FDOSS, by year (empirical)	

*CIDT, culture-independent diagnostic test; FDOSS, Foodborne Disease Outbreak Surveillance System; STEC, Shiga toxin-producing *Escherichia coli*.

Appendix 1 Table 4. Data sources (distribution) for the underdiagnosis multiplier of the surveillance data scaled-up model for illnesses associated with 6 pathogens*

	Underdiagnosis multiplier of illness, distribution		
Pathogen	Medical care seeking/specimen submission	% Laboratory test	Test sensitivity
Campylobacter spp.	Predicted using Bayesian models developed from the FoodNet Population Survey data (posterior)	FoodNet Laboratory Survey (empirical)	FoodNet reflex culture data (PERT)
STEC O157			1 (degenerate)
non-O157 STEC			1 (degenerate)
Nontyphoidal *Salmonella* spp. *serotypes*			FoodNet reflex culture data (PERT)
Listeria monocytogenes	Assumed to be high (PERT)	Assumed to be high (PERT)	Literature (PERT)
Clostridium perfringens	Multiplier for nontyphoidal *Salmonella* was used.		

*PERT, program evaluation review technique; STEC, Shiga toxin-producing *Escherichia coli*.

Appendix 1 Table 5. Total and outbreak-associated illnesses by pathogen during 2015–2019 in the FoodNet database*

Pathogen	Total no. laboratory-confirmed cases	No. outbreak-associated, laboratory-confirmed cases	Ratio
Yersinia enterocolitica	920	2	460.0:1
Campylobacter spp.	46,271	156	296.6:1
Salmonella enterica serotype Typhi	326	3	108.7:1
Cryptosporidium spp.	5,339	181	29.5:1
non-O157 STEC	8,108	292	27.7:1
Listeria monocytogenes	737	30	24.6:1
Vibrio spp.	1,860	83	22.4:1
Shigella spp.	13,129	687	19.1:1:1
Salmonella spp., nontyphoidal	41,704	2846	14.7:1
STEC O157	3,130	409	7.7:1
Cyclospora cayetanensis	1,376	389	3.5:1

STEC, Shiga toxin-producing *Escherichia coli*.

Appendix 1 Table 6. Data source (distribution) for underreporting, percent travel related, and percent foodborne of the surveillance data scaled-up model for 6 pathogens*

Pathogen	Underreporting	% Travel related	% Foodborne
Campylobacter spp.<br>STEC O157<br>non-O157 STEC<br>*Salmonella* nontyphoidal serotypes	1 (degenerate)	FoodNet data (PERT)	Structured expert judgment (empirical)
Listeria monocytogenes	1 (degenerate)	Listeria Initiative (PERT)	Assumed to be high (PERT)
Clostridium perfringens	FoodNet data (PERT)	Assumed to be low (PERT)	Assumed to be high (PERT)

*PERT, program evaluation review technique; STEC, Shiga toxin-producing *Escherichia coli*.

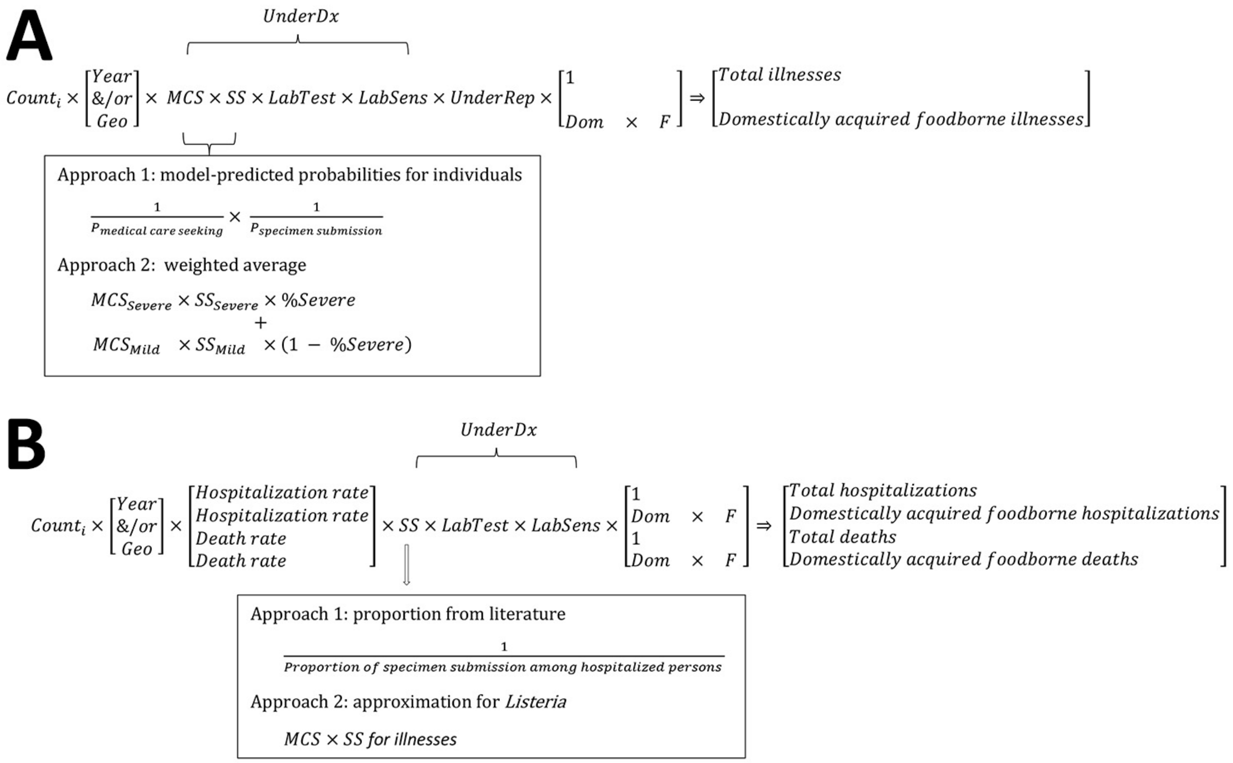

Appendix 1 Figure 1. Schematic illustration of surveillance data scaled-up model used to estimate illnesses, hospitalizations, and deaths according to numbers of laboratory-confirmed illnesses reported to surveillance. A) Estimates of illnesses; B) estimates of hospitalizations and deaths. $Count_i$ is the counts of laboratory-confirmed illnesses reported to disease surveillance. Year is a deterministic factor to standardize counts in given year to years 2017–2019. Geo is a deterministic factor to scale counts from the Foodborne Diseases Active Surveillance Network (FoodNet) sites up to the entire United States or equals 1 if counts are from a national disease surveillance system. UnderDx is an underdiagnosis multiplier that is the product of medical care seeking (MCS), specimen submission (SS), laboratory test (LabTest), and sensitivity of laboratory test (LabSens) factors. MCS is a factor to scale medical care seekers up to all illnesses with a Bayesian prediction model approach for FoodNet pathogens and severe and mild illness approach for invasive *Listeria monocytogenes*. In the Bayesian prediction model approach, MCS is the reciprocal of the predicted probability of medical care seeking (i.e., $\frac{1}{P_{medical\ care\ seeking}}$). A) SS is a factor to scale patients who submitted specimens up to all ill visits with a Bayesian prediction model approach for FoodNet pathogens and a severe illness approach for invasive *Listeria monocytogenes*. In the Bayesian prediction model approach, SS is the reciprocal of the predicted probability of specimen submission (i.e., $\frac{1}{P_{specimen\ submission}}$). B) SS is a factor to scale patients who submitted specimens up to all hospitalized patients with a reciprocal version for FoodNet pathogens (i.e., $\frac{1}{Proportion\ of\ specimen\ submission\ among\ hospitalized\ persons}$) and severe and mild illness approach for invasive *Listeria monocytogenes*. LabTest is a factor to scale tests performed up to specimens submitted.

LabSens is a factor to scale positive tests up to true positive specimens. UnderRep is a factor to scale illnesses reported to a passive surveillance or outbreak surveillance system up to illnesses reported to an active surveillance system (i.e., to harmonize counts from different types of surveillance for further common adjustment). Dom is a factor to scale total counts down to counts that are domestically acquired. F is a factor to scale counts down to counts that are foodborne.

A

$$U.S.Population \times Illness\ incidence\ rate \times UnderDx \times \begin{bmatrix} 1 \\ Dom \times F \end{bmatrix} \Rightarrow \begin{bmatrix} Total\ illnesses \\ Domestically\ acquired\ foodborne\ illnesses \end{bmatrix}$$

B

$$U.S.Population \times \begin{bmatrix} Hospitalization\ incidence\ rate \\ Hospitalization\ incidence\ rate \\ Death\ incidence\ rate \\ Death\ incidence\ rate \end{bmatrix} \times \begin{bmatrix} 1 \\ Dom \times F \\ 1 \\ Dom \times F \end{bmatrix} \Rightarrow \begin{bmatrix} Total\ hospitalizations \\ Domestically\ acquired\ foodborne\ hospitalizations \\ Total\ deaths \\ Domestically\ acquired\ foodborne\ illnesses \end{bmatrix}$$

Appendix 1 Figure 2. Schematic illustration of the population data scaled-down model used to estimate illnesses, hospitalizations, and deaths according to the US population size and disease incidence rates. A) Estimates of illnesses; B) estimates of hospitalizations and death. US Population refers to the population at risk (i.e., average of the 2017–2019 US census population). Incidence rate is new cases per year in a given population. UnderDx is underdiagnosis multiplier. For norovirus, we used an illness incidence rate from a publication indicating specimen submission, laboratory test, and test sensitivity. Thus, only medical care seeking was considered here. Dom is a factor to scale total counts down to counts that are domestically acquired. F is a factor to scale counts down to counts that are foodborne.

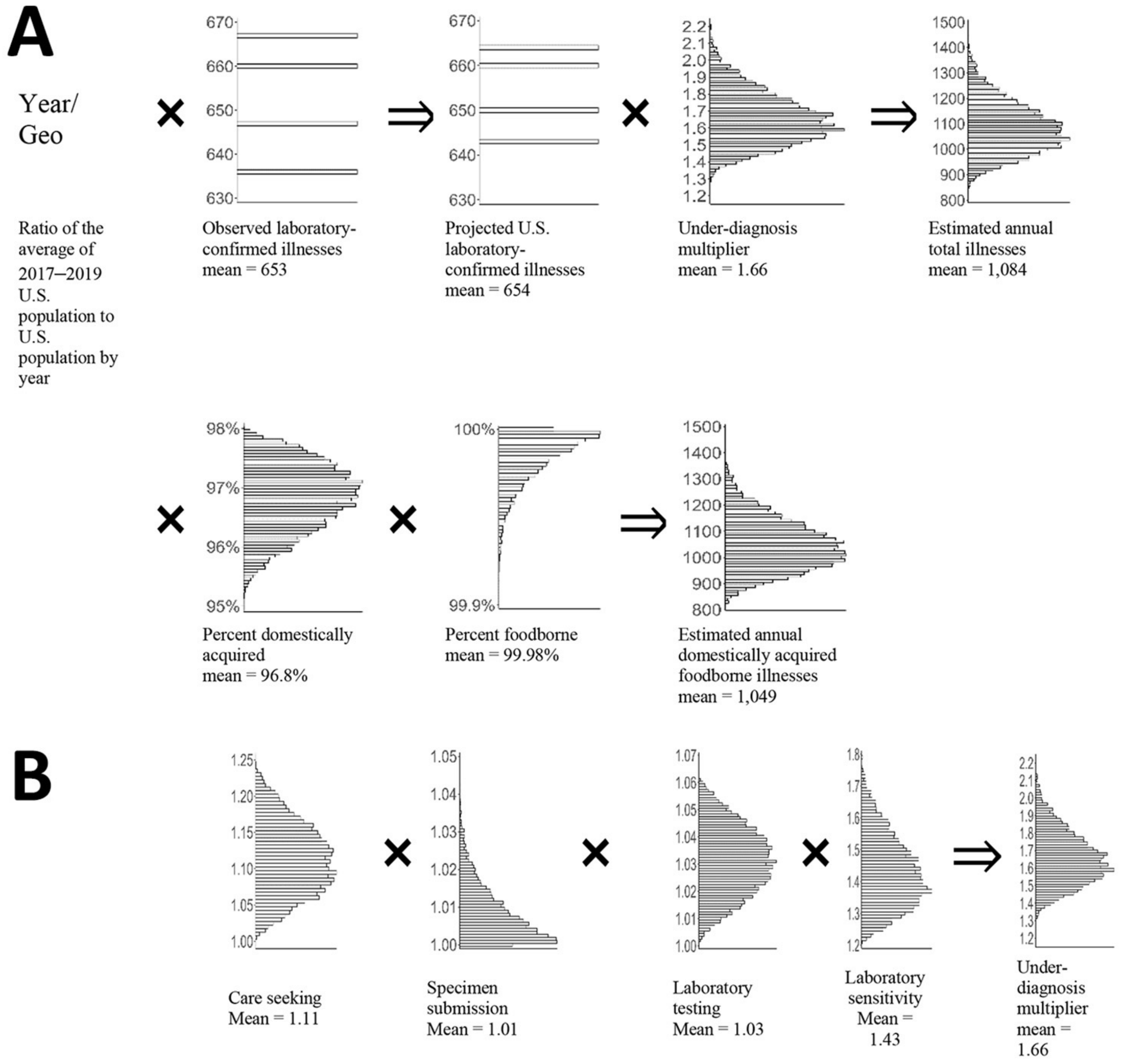

Appendix 1 Figure 3. Estimations of annual illnesses and underdiagnosis multiplier for *Listeria monocytogenes* in nonpregnant women. A) Estimation of annual illnesses. Histogram of observed laboratory-confirmed illness reflects annual counts reported to the *Listeria* Initiative during 2016–2019 adjusted for under-diagnosis, percent domestically acquired, and percent foodborne. We assumed there was no underreporting on the basis of illness severity. Year is a deterministic factor to standardize counts in a given year to the years 2017–2019. Geo denotes geography and is a deterministic factor to scale counts from the Foodborne Diseases Active Surveillance Network (FoodNet) sites up to the entire United States or equals 1 if counts are from a national disease surveillance system. B) Underdiagnosis multiplier for *Listeria* illnesses. The histograms represent the factors contributing to underdiagnosis (medical care seeking, specimen submission, laboratory testing, and laboratory test sensitivity).

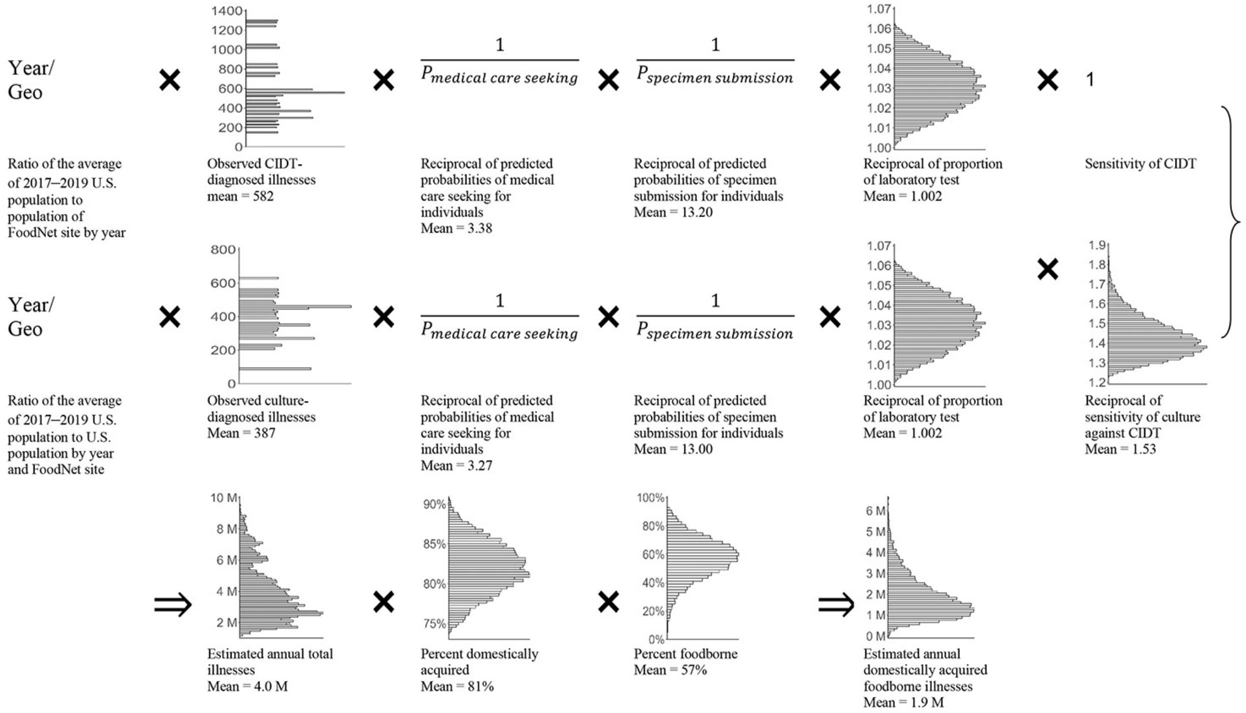

Appendix 1 Figure 4. Schematic diagram of the estimation of annual illnesses caused by *Campylobacter* spp. Histogram of observed laboratory-confirmed illnesses reflects annual counts reported by 10 FoodNet sites during 2017–2019. We assumed there was no underreporting because FoodNet is an active surveillance system. $P_{medical\ care\ seeking}$ and $P_{specimen\ submission}$ were computed for individual cases and implemented as degenerate distributions. Year is a deterministic factor to standardize counts in a given year to the years 2017–2019. Geo denotes geography and is a deterministic factor to scale counts from the Foodborne Diseases Active Surveillance Network (FoodNet) sites up to the entire United States or equals 1 if counts are from a national disease surveillance system. M, million.

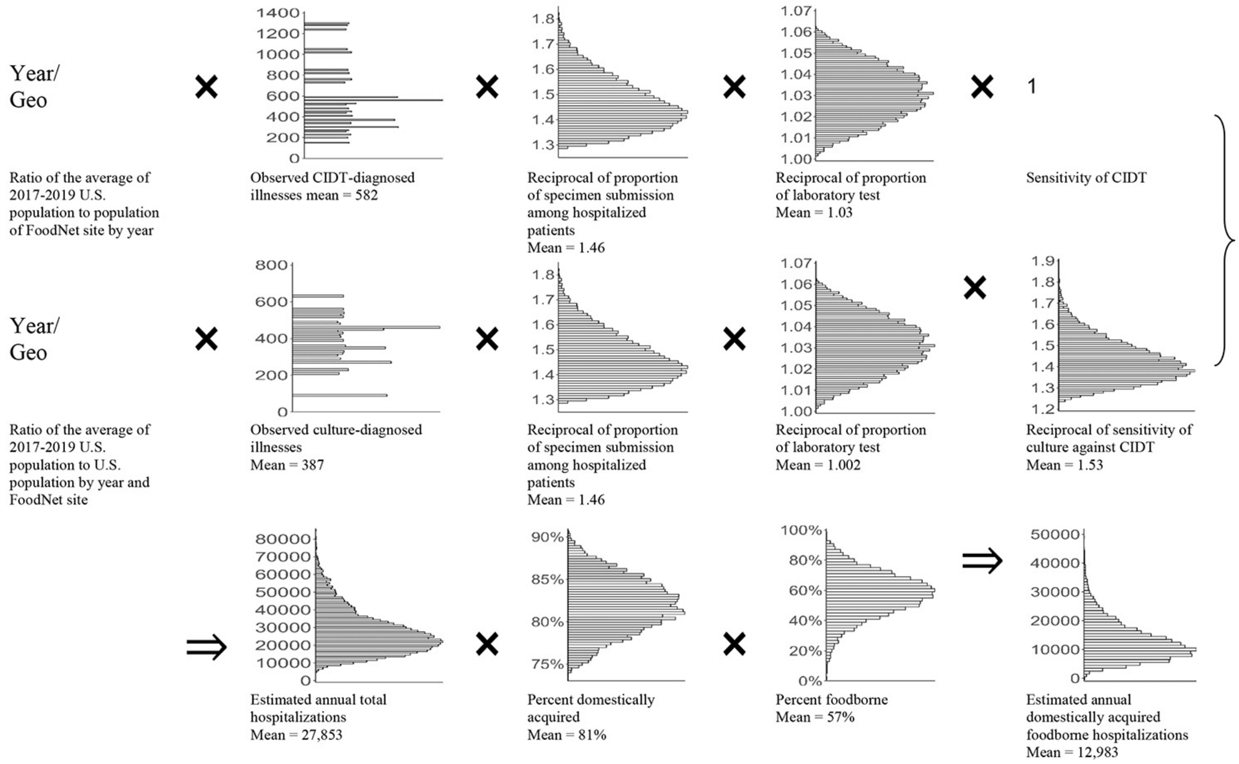

Appendix 1 Figure 5. Schematic diagram of the estimation of annual hospitalizations caused by *Campylobacter* spp. Histogram of observed laboratory-confirmed illness are annual counts reported by 10 FoodNet sites during 2017–2019. Year is a deterministic factor to standardize counts in a given year to the years 2017–2019. Geo denotes geography and is a deterministic factor to scale counts from the Foodborne Diseases Active Surveillance Network (FoodNet) sites up to the entire U.S. or equals to 1 if counts are from a national disease surveillance. M, million.

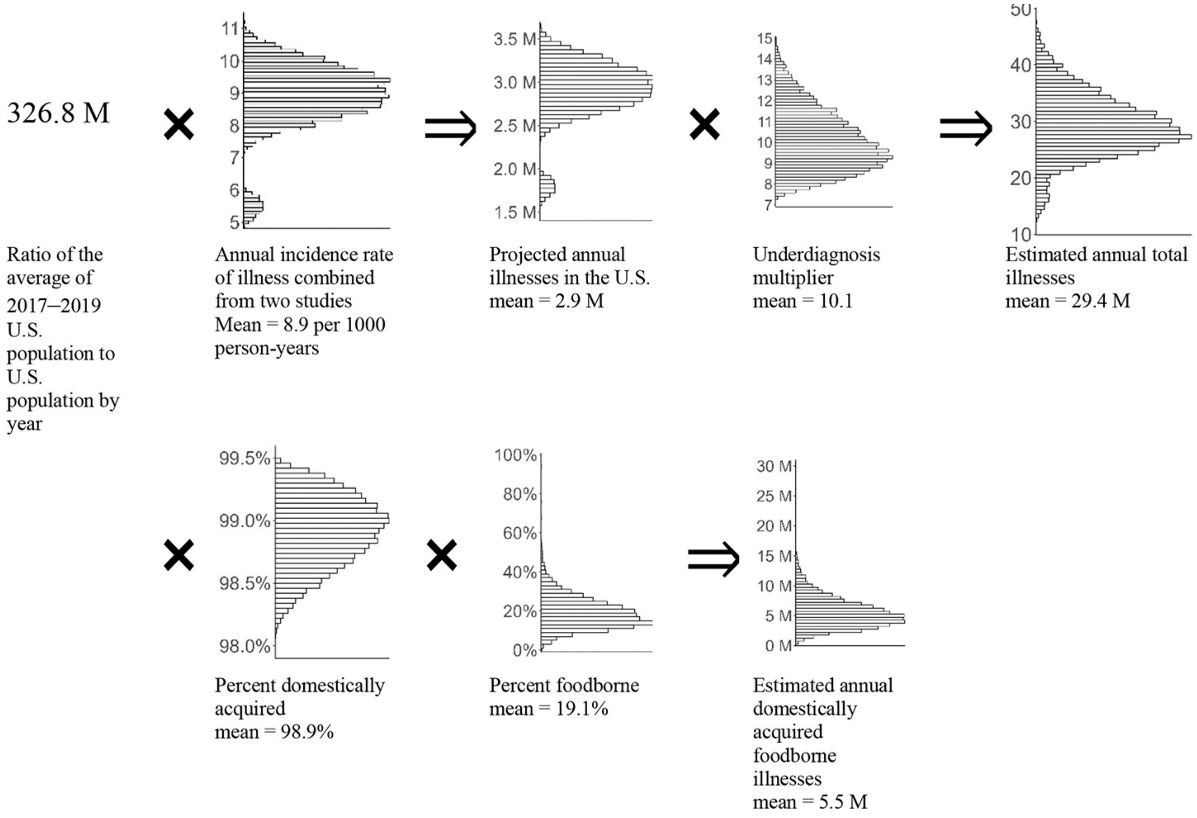

Appendix 1 Figure 6. Schematic diagram of the estimation of annual illnesses for norovirus. M, million.
