# Supplementary material for: Foodborne Illness Acquired in the United States—Major Pathogens, 2019
Source: Emerg Infect Dis. 2025 Apr;31(4):669–77. doi: 10.3201/eid3104.240913 (PMC11950263; doi:10.3201/eid3104.240913)
Supplement: Appendix 2 — Additional information for estimations and uncertainty model inputs used for foodborne illness acquired in the United States—major pathogens, 2019. [file 24-0913-Techapp-s2.pdf]

Article DOI: https://doi.org/10.3201/eid3104.240913
EID cannot ensure accessibility for supplementary materials supplied by authors. Readers who have difficulty accessing supplementary content should contact the authors for assistance.

Foodborne Illness Acquired in the United States—Major Pathogens, 2019

Appendix 2

Estimation and Uncertainty Model Inputs for 7 Major Pathogens Transmitted Through Food

Appendix 2 Table 1. *Campylobacter* spp.*

Model input	Data source(s)	Distribution	Distribution values
Reported illnesses	Number of laboratory-confirmed *Campylobacter* illnesses reported to the Foodborne Diseases Active Surveillance Network (FoodNet) by FoodNet site (n = 10) and year (2017–2019) (*1*).	Empirical	Minimum, lower quartile, median, upper quartile, maximum values: 562, 698, 915, 1222, 156.9 Refer to Appendix 2 Table 17 below for a full list of distribution values used.
Population adjustment by year	Population ratios applied to the number of laboratory-confirmed *Campylobacter* illnesses in each year and FoodNet site combination based on average 2017–2019 U.S. Census population estimates (*2*).	Degenerate	Minimum, lower quartile, median, upper quartile, maximum values: 30.7, 54.1, 77.1, 91.4, 156.1 Refer to Appendix 2 Table 18 below for a full list of distribution values used.
Underreporting	No underreporting multiplier. We assumed that all laboratory-confirmed illnesses were enumerated by FoodNet active surveillance.	NA	NA
Underdiagnosis			
Medical care seeking and specimen submission	Each laboratory-confirmed *Campylobacter* illness in FoodNet surveillance was adjusted for underdiagnosis due to medical care seeking and stool sample submission using a Bayesian approach based on the characteristics of people with acute diarrheal illness who reported seeking medical care and submitting a stool sample in the 2018–2019 FoodNet Population Survey (*3*). Characteristics included age group (<5, 5–64, 65+ years), sex, race and ethnicity (Hispanic; non-Hispanic Black; non-Hispanic white; non-Hispanic other race), and the presence fever and bloody diarrhea. Acute diarrheal illness was defined as diarrhea (≥3 loose stools in 24 h) lasting >1 d or resulting in restricted daily activities, excluding people who said that their illness was due to a long-lasting or chronic illness or condition, such as colitis or irritable bowel syndrome.	Posterior	Refer to Appendix 1
Laboratory testing	We accounted for the percentage of laboratories that routinely tested (on- or off-site) for *Campylobacter* using data from the FoodNet Laboratory Survey (*4*).	Empirical	Minimum, lower quartile, median, upper quartile, maximum values: 0.98, 1, 1, 1, 1

Model input	Data source(s)	Distribution	Distribution values
Test sensitivity	Separate adjustments were made for *Campylobacter* illnesses confirmed using culture and culture-independent diagnostic tests (CIDTs). We assumed that CIDTs were 100% sensitive and estimated the sensitivity of culture using reflex culture data from FoodNet. That is, sensitivity of culture was estimated as the proportion of positive reflex cultures among all reflex cultures in FoodNet, and assuming this to be a lower bound, this value was used as the lower parameter of the PERT distribution (refer to Appendix 1). Modal and high value were calculated by assuming that uncertainty was a 50% relative increase/decrease from modal on an odds scale.	PERT	Low, modal, high values (culture only): 0.56, 0.66, 0.74
Proportion hospitalized	Proportion of laboratory-confirmed *Campylobacter* illnesses reported to FoodNet that resulted in hospitalization by FoodNet site (n = 10) and year (2017–2019).	Empirical	Minimum, lower quartile, median, upper quartile, maximum values: 0.10, 0.15, 0.21, 0.25, 0.34 Refer to Appendix 2 Table 19 below for a full list of distribution values used.
Proportion who died	Proportion of laboratory-confirmed *Campylobacter* illnesses reported to FoodNet that resulted in death by FoodNet site (n = 10) and year (2017–2019).	Empirical	Minimum, lower quartile, median, upper quartile, maximum values: 0, 0.002, 0.003, 0.004, 0.010 Refer to Appendix 2 Table 20 below for a full list of distribution values used.
Specimen submission, hospitalizations and deaths	Proportion of hospitalized patients with nonspecific gastroenteritis diagnosis codes who submitted a stool sample for bacterial culture from two published studies (refer to Appendix 1) (*5*,*6*). Uncertainty with this proportion was based on a 50% relative increase/decrease on an odds scale.	PERT	Low, modal, high values: 0.61, 0.70, 0.78
Proportion travel-related	Proportion of laboratory-confirmed *Campylobacter* illnesses with reported travel outside the United States within 7 d of illness onset (2017–2019). Uncertainty with this proportion was based on a 50% relative increase/decrease on an odds scale.	PERT	Low, modal, high values: 0.13, 0.18, 0.25
Proportion foodborne	Proportion (mean and 95% uncertainty interval) of domestically acquired *Campylobacter* illnesses transmitted through food based on a structured expert judgment study (*7*).	Empirical	Minimum, lower quartile, median, upper quartile, maximum values: 0.10, 0.48, 0.68, 0.79, 0.95

*NA, not applicable; PERT, program evaluation and review technique.

Appendix 2 Table 2. *Clostridium perfingens**

Model input	Data source(s)	Distribution	Parameters
Reported illnesses	Number of *Clostridium perfingens* outbreak-associated illnesses reported to CDC's Foodborne Disease Outbreak Surveillance System (2010–2019) (*8*).	Empirical	By year (2010–2019): 1112, 392, 989, 382, 894, 686, 651, 965, 1475, 383
Population adjustment by year	Population ratios applied to each year from 2010–2019 based on average 2017–2019 U.S. Census population estimates (*2*).	Degenerate	Ratios by year (2010–2019): 1.06, 1.05, 1.04, 1.03, 1.03, 1.02, 1.01, 1.01, 1, 1
Underreporting	Outbreak surveillance underreporting multiplier used to adjust for underreporting (refer to Appendix 1).	PERT	Low, modal, high, [precision] values: 7, 25, 297, [64]
Underdiagnosis			
Medical care seeking and specimen submission	*Salmonella* non-typhoidal underdiagnosis multiplier applied.	NA	NA
Laboratory testing	*Salmonella* non-typhoidal underdiagnosis multiplier applied.	NA	NA
Test sensitivity	*Salmonella* non-typhoidal underdiagnosis multiplier applied.	NA	NA

Model input	Data source(s)	Distribution	Parameters
Proportion hospitalized	Proportion of outbreak-associated illnesses that resulted in a hospitalization in *Clostridium perfingens* outbreaks reported to the Foodborne Disease Outbreak Surveillance System (2010–2019).	Empirical	By year (2010–2019): 0.008, 0, 0.004, 0.005, 0.001, 0, 0.011, 0.047, 0.005, 0
Proportion who died	Proportion of outbreak-associated illnesses that resulted in a death in foodborne *Clostridium perfingens* outbreaks reported to the Foodborne Disease Outbreak Surveillance System (2010–2019).	Empirical	By year (2010–2019): 0.003, 0, 0, 0, 0, 0.001, 0.006, 0.001, 0, 0
Specimen submission, hospitalizations and deaths	Proportion of hospitalized patients with nonspecific gastroenteritis diagnosis codes who submitted a stool sample for bacterial culture from two published studies (refer to Appendix 1) (*5*,*6*). Uncertainty with this proportion was based on a 50% relative increase/decrease on an odds scale.	PERT	Low, modal, high values: 0.61, 0.70, 0.78
Proportion travel-related	Because of the rapid onset and short duration of illness caused by *Clostridium perfingens*, we assumed that almost 100% of illnesses occurring in the United States are domestically acquired.	PERT	Low, modal, high values: 0, 0, 0.02
Proportion foodborne	Estimates based on outbreak-associated illnesses from foodborne outbreaks reported to the Foodborne Disease Outbreak Surveillance System, therefore, estimated illnesses assumed to be 100% foodborne.	PERT	Low, modal, high values: 0.999, 1, 1

*NA, not applicable; PERT, program evaluation and review technique.

Appendix 2 Table 3. *Listeria monocytogenes*, nonpregnancy*

Model input	Data source(s)	Distribution	Distribution values
Reported illnesses	Number of invasive non-pregnancy-associated *Listeria monocytogenes* infections reported to CDC's *Listeria* Initiative (2016–2019) (*9*).	Empirical	By year (2016–2019): 639, 650, 661, 667
Population adjustment by year	Population ratios applied to each year from 2016–2019 based on average 2017–2019 U.S. Census population estimates (*2*).	Degenerate	Adjustment by year (2016–2019): 1.01,1.01,1,1
Underreporting	No underreporting multiplier. We assumed all diagnosed cases were reported because of the severity of invasive listeriosis; an assumption supported by similar reporting rates in FoodNet and non-FoodNet sites	NA	NA
Medical care seeking	Assumed to have a high rate of medical care seeking.	PERT	Low, modal, high values: 0.8, 0.9, 1
Specimen submission	Almost all cases assumed to submit a specimen for testing.	PERT	Low, modal, high values: 0.95, 1, 1
Laboratory testing	We assumed that most persons with listeriosis who submitted a specimen for testing would be tested for listeriosis.	PERT	Low, modal, high values: 0.94, 0.97, 1
Laboratory test sensitivity	71% based on published study of blood culture sensitivity.	PERT	Low, modal, high values: 0.55, 0.71, 0.83
Hospitalized	Number of invasive non-pregnancy-associated *Listeria monocytogenes* illnesses that resulted in hospitalization reported to CDC's *Listeria* Initiative (2016–2019).	Empirical	By year (2016–2019): 553, 571, 579, 585
Died	Number of invasive non-pregnancy-associated *Listeria monocytogenes* illnesses that resulted in death reported to CDC's *Listeria* Initiative (2016–2019).	Empirical	By year (2016–2019): 96, 103, 110, 105
Underdiagnosis for hospitalizations and deaths	Underdiagnosis based on the underdiagnosis multiplier for *Listeria monocytogenes* illnesses applied.	NA	NA
Proportion travel-related	Proportion of invasive *Listeria monocytogenes* illnesses reporting travel outside the United States within 30 d of illness onset (2016–2019). Uncertainty with this proportion was based on a 50% relative increase/decrease on an odds scale.	PERT	Low, modal, high values: 0.02, 0.03, 0.05
Proportion foodborne	Assumed to be almost 100% foodborne.	PERT	Low, modal, high values:

Model input	Data source(s)	Distribution	Distribution values
			0.999, 1, 1

*NA, not applicable; PERT, program evaluation and review technique.

Appendix 2 Table 4. *Listeria monocytogenes*, pregnancy (mothers)*

Model input	Data source(s)	Distribution	Distribution values
Reported illnesses	Number of invasive pregnancy-associated *Listeria monocytogenes* illnesses in mothers reported to CDC's Listeria Initiative (2016–2019) (*9*). Episodes of invasive illness in the mother were counted if *Listeria monocytogenes* was isolated from an invasive specimen source associated with the mother or products of conception or if the mother an invasive case reported symptoms.	Empirical	By year (2016–2019): 51, 72, 65, 77
Population adjustment by year	Population ratios applied to each year from 2016–2019 based on average 2017–2019 U.S. Census population estimates (*2*).	Degenerate	Adjustment by year (2016–2019): 1.01, 1.01, 1,1
Underreporting	No underreporting multiplier. We assumed all diagnosed cases were reported because of the severity of invasive listeriosis; an assumption supported by similar reporting rates in FoodNet and non-FoodNet sites.	NA	NA
Medical care seeking	Assumed to have a high rate of medical care seeking.	PERT	Low, modal, high values: 0.8, 0.9, 1
Specimen submission	Almost all cases assumed to submit a specimen for testing.	PERT	Low, modal, high values: 0.95, 1, 1
Laboratory testing	We assumed that most persons with listeriosis who submitted a specimen for testing would be tested for *Listeria monocytogenes*.	PERT	Low, modal, high values: 0.94, 0.97, 1
Laboratory test sensitivity	71% based on published study of blood culture sensitivity.	PERT	Low, modal, high values: 0.55, 0.71, 0.83
Hospitalized	Number of invasive pregnancy-associated *Listeria monocytogenes* illnesses in mothers that resulted in hospitalization reported to CDC's *Listeria* Initiative (2016–2019).	Empirical	By year (2016–2019): 39, 51, 45, 50
Died	Number of invasive pregnancy-associated Listeria monocytogenes illnesses in mothers that resulted in death reported to CDC's *Listeria* Initiative (2016–2019).	Empirical	By year (2016–2019): 0, 0, 0, 0
Underdiagnosis for hospitalizations and deaths	Underdiagnosis multiplier for *Listeria monocytogenes* illnesses applied	NA	NA
Proportion travel-related	Proportion of invasive *Listeria monocytogenes* illnesses reporting travel outside the United States within 30 d of illness onset (2016–2019). Uncertainty with this proportion was based on a 50% relative increase/decrease on an odds scale.	PERT	Low, modal, high values: 0.02, 0.03, 0.05
Proportion foodborne	Assumed to be almost 100% foodborne.	PERT	Low, modal, high values: 0.999, 1, 1

*NA, not applicable; PERT, program evaluation and review technique.

Appendix 2 Table 5. *Listeria monocytogenes*, pregnancy (infants, liveborn)*

Model input	Data source(s)	Distribution	Distribution values
Reported illnesses	Number of invasive pregnancy-associated *Listeria monocytogenes* illnesses in liveborn infants reported to CDC's *Listeria* Initiative (2016–2019) (*9*). Episodes of invasive illness in the infant were counted if *Listeria monocytogenes* was isolated from an invasive specimen source associated with the infant or products of conception or if the infant an invasive case reported symptoms.	Empirical	By year (2016–2019): 50, 54, 54, 71
Population adjustment by year	Population ratios applied to each year from 2016–2019 based on average 2017–2019 U.S. Census population estimates (*2*).	Degenerate	Adjustment by year (2016–2019): 1.01, 1.01, 1,1

Model input	Data source(s)	Distribution	Distribution values
Underreporting	No underreporting multiplier. We assumed all diagnosed cases were reported because of the severity of invasive listeriosis; an assumption supported by similar reporting rates in FoodNet and non-FoodNet sites.	NA	NA
Medical care seeking	Assumed to have a high rate of medical care seeking.	PERT	Low, modal, high values: 0.8, 0.9, 1
Specimen submission	Almost all cases assumed to submit a specimen for testing.	PERT	Low, modal, high values: 0.95, 1, 1
Laboratory testing	We assumed that most persons with listeriosis who submitted a specimen for testing would be tested for *Listeria monocytogenes*.	PERT	Low, modal, high values: 0.94, 0.97, 1
Laboratory test sensitivity	71% based on published study of blood culture sensitivity.	PERT	Low, modal, high values: 0.55, 0.71, 0.83
Hospitalized	Number of invasive pregnancy-associated *Listeria monocytogenes* infections in liveborn infants that resulted in hospitalization reported to CDC's Listeria Initiative who were hospitalized (2016–2019).	Empirical	By year (2016–2019): 44, 41, 40, 60
Died	Number of invasive pregnancy-associated Listeria monocytogenes illnesses in liveborn infants that resulted in death reported to CDC's *Listeria* Initiative (2016–2019).	Empirical	By year (2016–2019): 0, 5, 3, 6
Underdiagnosis, hospitalizations and deaths	Underdiagnosis multiplier for *Listeria monocytogenes* illnesses applied	NA	NA
Proportion travel-related	Proportion of invasive *Listeria monocytogenes* illnesses reporting travel outside the United States within 30 d of illness onset (2016–2019). Uncertainty with this proportion was based on a 50% relative increase/decrease on an odds scale.	PERT	Low, modal, high values: 0.02, 0.03, 0.05
Proportion foodborne	Assumed to be almost 100% foodborne.	PERT	Low, modal, high values: 0.999, 1, 1

*NA, not applicable; PERT, program evaluation and review technique.

Appendix 2 Table 6. *Listeria monocytogenes*, pregnancy (fetal deaths)*

Model input	Data source(s)	Distribution	Distribution values
Number of fetal deaths	Number of invasive pregnancy-associated *Listeria monocytogenes* illnesses resulting in fetal deaths reported to CDC's *Listeria* Initiative (2016–2019) (*9*).	Empirical	By year (2016–2019): 18, 25, 25, 24
Population adjustment by year	Population ratios applied to each year from 2016–2019 based on average 2017–2019 U.S. Census population estimates (*2*).	Degenerate	Adjustment by year (2016–2019): 1.01, 1.01, 1, 1
Underdiagnosis, hospitalizations and deaths	Underdiagnosis based on the underdiagnosis multiplier for *Listeria monocytogenes* illnesses.	NA	NA
Proportion travel-related	Proportion of invasive *Listeria monocytogenes* illnesses reporting travel outside the United States within 30 d of illness onset (2016–2019). Uncertainty with this proportion was based on a 50% relative increase/decrease on an odds scale.	PERT	Low, modal, high values: 0.02, 0.03, 0.05
Proportion foodborne	Assumed to be almost 100% foodborne.	PERT	Low, modal, high values: 0.999, 1, 1

*NA, not applicable; PERT, program evaluation and review technique.

Appendix 2 Table 7. Norovirus*

Model input	Data source(s)	Distribution	Parameters
Reported illnesses	Incidence of norovirus illnesses from two studies applied to the average 2017–2019 U.S. census population. The first study conducted active surveillance among all enrolled members of Kaiser Permanente Northwest in the Portland, Oregon, metropolitan area from 2014–2016. The estimated incidence of medically attended norovirus was per 5.5 per 1000 person-years (95% CI 4.8–6.1)] (*10*); the second study used the IBM MarketScan Commercial and Medicare	-	See Data source(s) description.

Model input	Data source(s)	Distribution	Parameters
	Supplemental Databases from 2001–2015 to estimate the number of ambulatory clinic visits (74.9 (95% CI: 57.5–95.3) and emergency department visits (15.3, 95% CI: 11.5–20.1) due to norovirus per 1000 person-years (*11*). Data from the same study were combined by adding up the PERT distributions constructed with the point estimate as modal and confidence limits as low and high values. Data from the two studies were combined using a random sampling from the two constructed distributions with weights proportional to the number of annual cases reported in the two studies.		
Population at risk	Incidence from combined studies applied to average 2017–2019 U.S. Census population estimates (*2*).	Degenerate	326, 763, 427
Underreporting	Norovirus estimates were not adjusted for under-reporting because this was accounted for in the source data.	NA	NA
Underdiagnosis			
Medical care seeking and specimen submission	We adjusted for underdiagnosis by estimating the percentage of 2018–2019 FoodNet survey respondents with acute gastroenteritis (defined as diarrhea ≥3 loose stools in a 24-h period beginning within the past month) lasting <3 d who sought medical care. Like previous studies, (Hall et al. 2011) we used <3 d because viral diarrhea is generally of shorter duration than diarrhea of other etiologies. Because both papers (*10*,*11*) estimated illnesses among persons seeking medical care who submitted a stool specimen, no further adjustment was made for stool sample submission.	PERT	Low, modal, high values: 0.06, 0.10, 0.14
Laboratory testing	No adjustment made because this was accounted for in the source data.	NA	NA
Test sensitivity	No adjustment made because this was accounted for in the source data.	NA	NA
Incidence of hospitalization	Incidence of norovirus hospitalizations (10,000 person-years) estimated using the Healthcare Utilization Project National Inpatient Sample applied to the average 2017–2019 U.S. Census population (*11*).	PERT	Low, modal, high values: 2.6, 3.6, 4.8
Incidence of deaths	Incidence of norovirus deaths (10,000 person-years) was estimated using the National Center for Health Statistics multiple-cause-of-mortality data, applied to the average 2017–2019 U.S. Census population (*11*).	PERT	Low, modal, high values: 2.2, 2.8, 3.6
Proportion traveled	Assumed to be low within the incubation period for norovirus.	PERT	Low, modal, high values: 0.005, 0.01, 0.02
Proportion foodborne	Proportion (mean and 95% uncertainty interval) of domestically acquired illnesses transmitted through food based on a structured expert judgment study (*7*).	Empirical	Minimum, lower quartile, median, upper quartile, maximum values: 0.03, 0.12, 0.17, 0.24, 0.50

*NA, not applicable; PERT, program evaluation and review technique.

Appendix 2 Table 8. Nontyphoidal *Salmonella* serotype Enteritidis*

Model input	Data source(s)	Distribution	Distribution values
Reported illnesses	Number of laboratory-confirmed *S.* Enteritidis illnesses reported to the Foodborne Diseases Active Surveillance Network (FoodNet) by FoodNet site (n = 10) and year (2017–2019) (*1*). *Salmonella* serotypes with serotype not identified were randomly assigned to one of the six serotype groups with weights equal to the proportions of isolates with known serotype (refer to Appendix 1).	Empirical	Minimum, lower quartile, median, upper quartile, maximum values: 46, 94, 120, 213, 428 Refer to Appendix 2 Table 17 below for a full list of distribution values.
Population adjustment by year	Population ratios applied to the number of laboratory-confirmed *S.* Enteritidis illnesses in each year and FoodNet site combination based on average 2017–2019 U.S. Census population estimates (*2*).	Degenerate	Minimum, lower quartile, median, upper quartile, maximum values: 30.7, 54.1, 77.1, 91.4, 156.1 Refer to Appendix 2 Table 18 below for a full list of distribution values used.

Model input	Data source(s)	Distribution	Distribution values
Underreporting	No underreporting multiplier. We assumed that all laboratory-confirmed illnesses were enumerated by FoodNet active surveillance.	NA	NA
Underdiagnosis			
Medical care seeking specimen submission	Each laboratory-confirmed *S.* Enteritidis illness in FoodNet surveillance was adjusted for underdiagnosis due to medical care seeking and stool sample submission based on the characteristics of people with acute diarrheal illness who reported seeking medical care and submitting a stool sample in the 2018–2019 FoodNet Population Survey (*3*). Characteristics included age group (<5, 5–64, 65+ years), sex, race and ethnicity (Hispanic; non-Hispanic Black; non-Hispanic white; non-Hispanic other race), and the presence fever and bloody diarrhea. Acute diarrheal illness was defined as diarrhea (≥3 loose stools in 24 h) lasting >1 d or resulting in restricted daily activities, excluding people who said that their illness was due to a long-lasting or chronic illness or condition, such as colitis or irritable bowel syndrome.	Posterior	Refer to Appendix 1
Laboratory testing	We accounted for the percentage of laboratories that routinely tested (on-or off-site) for nontyphoidal *Salmonella* using data from the FoodNet Laboratory Survey (*4*).	Empirical	Minimum, lower quartile, median, upper quartile, maximum values: 0.98, 1, 1, 1, 1
Test sensitivity	Separate adjustments were made for nontyphoidal *Salmonella* illnesses confirmed using culture and culture-independent diagnostic tests (CIDTs). We assumed that CIDTs were 100% sensitive and estimated the sensitivity of culture using reflex culture data from FoodNet. That is, sensitivity of culture was estimated as the proportion of positive reflex cultures among all reflex cultures in FoodNet, and assuming this to be a lower bound, this value was used as the lower parameter of the PERT distribution (Appendix 1).	PERT	Low, modal, high values (culture only): 0.86, 0.90, 0.93
Proportion hospitalized	Proportion of laboratory-confirmed *S.* Enteritidis illnesses reported to FoodNet that resulted in hospitalization by FoodNet site (n = 10) and year (2017–2019).	Empirical	Minimum, lower quartile, median, upper quartile, maximum values: 0.15, 0.23, 0.27, 0.33, 0.41 Refer to Appendix 2 Table 19 below for a full list of distribution values used.
Proportion who died	Proportion of laboratory-confirmed *S.* Enteritidis illnesses reported to FoodNet that resulted in death by FoodNet site (n = 10) and year (2017–2019).	Empirical	Minimum, lower quartile, median, upper quartile, maximum values: 0, 0, 0, 0.010, 0.031 Refer to Appendix 2 Table 20 below for a full list of distribution values used.
Specimen submission, hospitalizations and deaths	Proportion of hospitalized patients with nonspecific gastroenteritis diagnosis codes who submitted a stool sample for bacterial culture from two published studies (Refer to Appendix 1) (*5*,*6*). Uncertainty with this proportion was based on a 50% relative increase/decrease on an odds scale.	PERT	Low, modal, high values: 0.61, 0.70, 0.78
Proportion travel-related	Proportion of laboratory-confirmed *S.* Enteritidis illnesses with reported travel outside the United States within 7 d of illness onset (2017–2019). Uncertainty with this proportion was based on a 50% relative increase/decrease on an odds scale.	PERT	Low, modal, high values: 0.16, 0.22, 0.30
Proportion foodborne	Proportion (mean and 95% uncertainty interval) of domestically acquired *S.* Enteritidis illnesses transmitted through food based on a structured expert judgment study (*7*).	Empirical	Minimum, lower quartile, median, upper quartile, maximum values: 0.40, 0.79, 0.90, 0.93, 1.00

*NA, not applicable; PERT, program evaluation and review technique.

Appendix 2 Table 9. Nontyphoidal *Salmonella* serotype I 4,[5],12:i:-*

Model input	Data source(s)	Distribution	Distribution values
Reported illnesses	Number of laboratory-confirmed *S.* I 4,[5],12:i:- illnesses reported to the Foodborne Diseases Active Surveillance Network (FoodNet) by FoodNet site (n = 10) and year (2017–2019) (*1*). *Salmonella* serotypes with serotype not identified were randomly assigned to one of the six serotype groups with weights equal to the proportions of isolates with known serotype (refer to Appendix 1).	Empirical	Minimum, lower quartile, median, upper quartile, maximum values: 16, 29, 43, 68, 125 Refer to Appendix 2 Table 17 below for a full list of distribution values.
Population adjustment by year	Population ratios applied to the number of laboratory-confirmed *S.* I 4,[5],12:i:- illnesses in each year and FoodNet site combination based on average 2017–2019 U.S. Census population estimates (*2*).	Degenerate	Minimum, lower quartile, median, upper quartile, maximum values: 30.7, 54.1, 77.1, 91.4, 156.1 Refer to Appendix 2 Table 18 below for a full list of distribution values used.
Underreporting	No underreporting multiplier. We assumed that all laboratory-confirmed illnesses were enumerated by FoodNet active surveillance.	NA	NA
Underdiagnosis			
Medical care seeking and specimen submission	Each laboratory-confirmed *S.* I 4,[5],12:i:- illnesses in FoodNet surveillance was adjusted for underdiagnosis due to medical care seeking and stool sample submission based on the characteristics of people with acute diarrheal illness who reported seeking medical care and submitting a stool sample in the 2018–2019 FoodNet Population Survey (*3*). Characteristics included age group (<5, 5–64, 65+ years), sex, race and ethnicity (Hispanic; non-Hispanic Black; non-Hispanic white; non-Hispanic other race), and the presence fever and bloody diarrhea. Acute diarrheal illness was defined as diarrhea (≥3 loose stools in 24 h) lasting >1 d or resulting in restricted daily activities, excluding people who said that their illness was due to a long-lasting or chronic illness or condition, such as colitis or irritable bowel syndrome.	Posterior	Refer to Appendix 1
Laboratory testing	We accounted for the percentage of laboratories that routinely tested (on- or off-site) for nontyphoidal *Salmonella* using data from the FoodNet Laboratory Survey (*4*).	Empirical	Minimum, lower quartile, median, upper quartile, maximum values:0.98, 1, 1, 1, 1
Test sensitivity	Separate adjustments were made for nontyphoidal *Salmonella* illnesses confirmed using culture and culture-independent diagnostic tests (CIDTs). We assumed that CIDTs were 100% sensitive and estimated the sensitivity of culture using reflex culture data from FoodNet. That is, sensitivity of culture was estimated as the proportion of positive reflex cultures among all reflex cultures in FoodNet, and assuming this to be a lower bound, this value was used as the lower parameter of the PERT distribution (refer to Appendix 1).	PERT	Low, modal, high values (culture only): 0.88, 0.92, 0.95
Proportion hospitalized	Proportion of laboratory-confirmed *S.* I 4,[5],12:i:- illnesses reported to FoodNet that resulted in hospitalization by FoodNet site (n = 10) and year (2017–2019).	Empirical	Minimum, lower quartile, median, upper quartile, maximum values: 0.10, 0.21, 0.29, 0.36, 0.49 Refer to Appendix 2 Table 19 below for a full list of distribution values used.
Proportion who died	Proportion of laboratory-confirmed *S.* I 4,[5],12:i:- illnesses reported to FoodNet that resulted in death by FoodNet site (n = 10) and year (2017–2019).	Empirical	Minimum, lower quartile, median, upper quartile, maximum values: 0, 0, 0, 0, 0.077 Refer to Appendix 2 Table 20 below for a full list of distribution values used.
Specimen submission, hospitalizations and deaths	Proportion of hospitalized patients with nonspecific gastroenteritis diagnosis codes who submitted a stool sample for bacterial culture from two published studies (refer to Appendix 1)	PERT	Low, modal, high values: 0.61, 0.70, 0.78

Model input	Data source(s)	Distribution	Distribution values
	(*5*,*6*). Uncertainty with this proportion was based on a 50% relative increase/decrease on an odds scale.		
Proportion travel-related	Proportion of laboratory-confirmed *Salmonella* nontyphoidal illnesses with reported travel outside the United States within 7 d of illness onset (2017–2019). Uncertainty with this proportion was based on a 50% relative increase/decrease on an odds scale.	PERT	Low, modal, high values: 0.07, 0.10, 0.14
Proportion foodborne	Proportion of domestically acquired *S.* I 4,[5],12:i:- illnesses transmitted through food based on a structured expert judgment study (*7*).	Empirical	Minimum, lower quartile, median, upper quartile, maximum values: 0, 0.64, 0.70, 0.81, 0.95

*NA, not applicable; PERT, program evaluation and review technique.

Appendix 2 Table 10. Nontyphoidal *Salmonella* serotype Javiana*

Model input	Data source(s)	Distribution	Distribution values
Reported illnesses	Number of laboratory-confirmed *S.* Javiana illnesses reported to the Foodborne Diseases Active Surveillance Network (FoodNet) by FoodNet site (n = 10) and year (2017–2019) (*1*). *Salmonella* serotypes with serotype not identified were randomly assigned to one of the six serotype groups with weights equal to the proportions of isolates with known serotype (refer to Appendix 1).	Empirical	Minimum, lower quartile, median, upper quartile, maximum values: 12, 17, 24, 88, 484 Refer to Appendix 2 Table 17 below for a full list of distribution values.
Population adjustment by year	Population ratios applied to the number of laboratory-confirmed for nontyphoidal *Salmonella* illnesses in each year and FoodNet site combination based on average 2017–2019 U.S. Census population estimates (*2*).	Degenerate	Minimum, lower quartile, median, upper quartile, maximum values: 30.7, 54.1, 77.1, 91.4, 156.1 Refer to Appendix 2 Table 18 below for a full list of distribution values used.
Underreporting	No underreporting multiplier. We assumed that all laboratory-confirmed illnesses were enumerated by FoodNet active surveillance.	NA	NA
Underdiagnosis			
Medical care seeking and specimen submission	Each laboratory-confirmed for nontyphoidal *Salmonella* illnesses in FoodNet surveillance was adjusted for underdiagnosis due to medical care seeking and stool sample submission based on the characteristics of people with acute diarrheal illness who reported seeking medical care and submitting a stool sample in the 2018–2019 FoodNet Population Survey (*3*). Characteristics included age group (<5, 5–64, 65+ years), sex, race and ethnicity (Hispanic; non-Hispanic Black; non-Hispanic white; non-Hispanic other race), and the presence fever and bloody diarrhea. Acute diarrheal illness was defined as diarrhea (≥3 loose stools in 24 h) lasting >1 d or resulting in restricted daily activities, excluding people who said that their illness was due to a long-lasting or chronic illness or condition, such as colitis or irritable bowel syndrome.	Posterior	Refer to Appendix 1
Laboratory testing	We accounted for the percentage of laboratories that routinely tested (on- or off-site) for nontyphoidal *Salmonella* using data from the FoodNet Laboratory Survey (*4*).	Empirical	Minimum, lower quartile, median, upper quartile, maximum values:0.98, 1, 1, 1, 1
Test sensitivity	Separate adjustments were made for nontyphoidal *Salmonella* illnesses confirmed using culture and culture-independent diagnostic tests (CIDTs). We assumed that CIDTs were 100% sensitive and estimated the sensitivity of culture using reflex culture data from FoodNet. That is, sensitivity of culture was estimated as the proportion of positive reflex cultures among all reflex cultures in FoodNet, and assuming this to be a lower bound, this value was used as the lower parameter of the PERT distribution (refer to Appendix 1).	PERT	Low, modal, high values (culture only): 0.82, 0.87, 0.91
Proportion hospitalized	Proportion of laboratory-confirmed S. Javiana illnesses reported to FoodNet that resulted in hospitalization by FoodNet site (n = 10) and year (2017–2019).	Empirical	Minimum, lower quartile, median, upper quartile, maximum values:

Model input	Data source(s)	Distribution	Distribution values
			0, 0.17, 0.24, 0.31, 0.42<br>Refer to Appendix 2 Table 19 below for a full list of distribution values used.
Proportion who died	Proportion of laboratory-confirmed *S.* Javiana illnesses reported to FoodNet that resulted in death by FoodNet site (n = 10) and year (2017–2019).	Empirical	Minimum, lower quartile, median, upper quartile, maximum values:<br>0, 0, 0, 0, 0.033<br>Refer to Appendix 2 Table 20 below for a full list of distribution values used.
Specimen submission, hospitalizations and deaths	Proportion of hospitalized patients with nonspecific gastroenteritis diagnosis codes who submitted a stool sample for bacterial culture from two published studies (refer to Appendix 1) (*5*,*6*). Uncertainty with this proportion was based on a 50% relative increase/decrease on an odds scale.	PERT	Low, modal, high values:<br>0.61, 0.70, 0.78
Proportion travel-related	Proportion of laboratory-confirmed *S.* Javiana illnesses with reported travel outside the United States within 7 d of onset (2017–2019). Uncertainty with this proportion was based on a 50% relative increase/decrease on an odds scale.	PERT	Low, modal, high values:<br>0.03, 0.05, 0.07
Proportion foodborne	Proportion of domestically acquired *S.* Javiana illnesses transmitted through food based on a structured expert judgment study (*7*).	Empirical	Minimum, lower quartile, median, upper quartile, maximum values:<br>0, 0.46, 0.60, 0.74, 0.90

*NA, not applicable; PERT, program evaluation and review technique.

Appendix 2 Table 11. Nontyphoidal *Salmonella* serotype Newport*

Model input	Data source(s)	Distribution	Distribution values
Reported illnesses	Number of laboratory-confirmed *S.* Newport illnesses reported to the Foodborne Diseases Active Surveillance Network (FoodNet) by FoodNet site (n = 10) and year (2017–2019) (*1*). *Salmonella* serotypes with serotype not identified were randomly assigned to one of the six serotype groups with weights equal to the proportions of isolates with known serotype (refer to Appendix 1).	Empirical	Minimum, lower quartile, median, upper quartile, maximum values:<br>24, 35, 51, 98, 423<br>Refer to Appendix 2 Table 17 below for a full list of distribution values.
Population adjustment (year)	Population ratios applied to the number of laboratory-confirmed nontyphoidal *Salmonella* illnesses in each year and FoodNet site combination based on average 2017–2019 U.S. Census population estimates (*2*).	Degenerate	Minimum, lower quartile, median, upper quartile, maximum values:<br>30.7, 54.1, 77.1, 91.4, 156.1<br>Refer to Appendix 2 Table 18 below for a full list of distribution values used.
Underreporting	No underreporting multiplier. We assumed that all laboratory-confirmed illnesses were enumerated by FoodNet active surveillance.	NA	NA
Underdiagnosis			
Medical care seeking and specimen submission	Each laboratory-confirmed nontyphoidal *Salmonella* illnesses in FoodNet surveillance was adjusted for underdiagnosis due to medical care seeking and stool sample submission based on the characteristics of people with acute diarrheal illness who reported seeking medical care and submitting a stool sample in the 2018–2019 FoodNet Population Survey (*3*). Characteristics included age group (<5, 5–64, 65+ years), sex, race and ethnicity (Hispanic; non-Hispanic Black; non-Hispanic white; non-Hispanic other race), and the presence fever and bloody diarrhea. Acute diarrheal illness was defined as diarrhea (≥3 loose stools in 24 h) lasting >1 d or resulting in restricted daily activities, excluding people who said that their illness was due to a long-lasting or chronic illness or condition, such as colitis or irritable bowel syndrome.	Posterior	Refer to Appendix 1

Model input	Data source(s)	Distribution	Distribution values
Laboratory testing	We accounted for the percentage of laboratories that routinely tested (on- or off-site) for *S.* Newport using data from the FoodNet Laboratory Survey (*4*).	Empirical	Minimum, lower quartile, median, upper quartile, maximum values:0.98, 1, 1, 1, 1
Test sensitivity	Separate adjustments were made for nontyphoidal *Salmonella* illnesses confirmed using culture and culture-independent diagnostic tests (CIDTs). We assumed that CIDTs were 100% sensitive and estimated the sensitivity of culture using reflex culture data from FoodNet. That is, sensitivity of culture was estimated as the proportion of positive reflex cultures among all reflex cultures in FoodNet, and assuming this to be a lower bound, this value was used as the lower parameter of the PERT distribution (refer to Appendix 1).	PERT	Low, modal, high values (culture only): 0.87, 0.91, 0.94
Proportion hospitalized	Proportion of laboratory-confirmed *S.* Newport illnesses reported to FoodNet that resulted in hospitalization by FoodNet site (n = 10) and year (2017–2019).	Empirical	Minimum, lower quartile, median, upper quartile, maximum values: 0.10, 0.20, 0.27, 0.33, 0.46 Refer to Appendix 2 Table 19 below for a full list of distribution values used.
Proportion who died	Proportion of laboratory-confirmed *S.* Newport illnesses reported to FoodNet that resulted in death by FoodNet site (n = 10) and year (2017–2019).	Empirical	Minimum, lower quartile, median, upper quartile, maximum values: 0, 0, 0, 0, 0.031 Refer to Appendix 2 Table 20 below for a full list of distribution values used.
Specimen submission (Hospitalizations, deaths)	Proportion of hospitalized patients with nonspecific gastroenteritis diagnosis codes who submitted a stool sample for bacterial culture from two published studies (refer to Appendix 1) (*5*,*6*). Uncertainty with this proportion was based on a 50% relative increase/decrease on an odds scale.	PERT	Low, modal, high values: 0.61, 0.70, 0.78
Proportion travel-related	Proportion of laboratory-confirmed *S.* Newport illnesses with reported travel outside the United States within 7 d of illness onset (2017–2019). Uncertainty with this proportion was based on a 50% relative increase/decrease on an odds scale.	PERT	Low, modal, high values: 0.05, 0.07, 0.10
Proportion foodborne	Proportion of domestically acquired *S.* Newport illnesses transmitted through food based on a structured expert judgment study (*7*).	Empirical	Minimum, lower quartile, median, upper quartile, maximum values: 0, 0.74, 0.80, 0.88, 0.95

*NA, not applicable; PERT, program evaluation and review technique.

Appendix 2 Table 12. Nontyphoidal *Salmonella* serotype Typhimurium*

Model input	Data source(s)	Distribution	Distribution values
Reported illnesses	Number of laboratory-confirmed *S.* Typhimurium illnesses reported to the Foodborne Diseases Active Surveillance Network (FoodNet) by FoodNet site (n = 10) and year (2017–2019) (*1*). *Salmonella* serotypes with serotype not identified were randomly assigned to one of the six serotype groups with weights equal to the proportions of isolates with known serotype (refer to Appendix 1).	Empirical	Minimum, lower quartile, median, upper quartile, maximum values: 35, 54, 68, 103, 212 Refer to Appendix 2 Table 17 below for a full list of distribution values.
Population adjustment by year	Population ratios applied to the number of laboratory-confirmed *S.* Typhimurium illnesses in each year and FoodNet site combination based on average 2017–2019 U.S. Census population estimates (*2*).	Degenerate	Minimum, lower quartile, median, upper quartile, maximum values: 30.7, 54.1, 77.1, 91.4, 156.1 Refer to Appendix 2 Table 18 below for a full list of distribution values used.

Model input	Data source(s)	Distribution	Distribution values
Underreporting	No underreporting multiplier. We assumed that all laboratory-confirmed illnesses were enumerated by FoodNet active surveillance.	NA	NA
Underdiiagnosis			
Medical care seeking and specimen submission	Each laboratory-confirmed *S.* Typhimurium illness in FoodNet surveillance was adjusted for underdiagnosis due to medical care seeking and stool sample submission based on the characteristics of people with acute diarrheal illness who reported seeking medical care and submitting a stool sample in the 2018–2019 FoodNet Population Survey (*3*). Characteristics included age group (<5, 5–64, 65+ years), sex, race and ethnicity (Hispanic; non-Hispanic Black; non-Hispanic white; non-Hispanic other race), and the presence fever and bloody diarrhea. Acute diarrheal illness was defined as diarrhea (≥3 loose stools in 24 h) lasting >1 d or resulting in restricted daily activities, excluding people who said that their illness was due to a long-lasting or chronic illness or condition, such as colitis or irritable bowel syndrome.	Posterior	Refer to Appendix 1
Laboratory testing	We accounted for the percentage of laboratories that routinely tested (on- or off-site) for nontyphoidal *Salmonella* using data from the FoodNet Laboratory Survey (*4*).	Empirical	Minimum, lower quartile, median, upper quartile, maximum values:0.98, 1, 1, 1, 1
Test sensitivity	Separate adjustments were made for nontyphoidal *Salmonella illnesses* confirmed using culture and culture-independent diagnostic tests (CIDTs). We assumed that CIDTs were 100% sensitive and estimated the sensitivity of culture using reflex culture data from FoodNet. That is, sensitivity of culture was estimated as the proportion of positive reflex cultures among all reflex cultures in FoodNet, and assuming this to be a lower bound, this value was used as the lower parameter of the PERT distribution (refer to Appendix 1).	PERT	Low, modal, high values (culture only): 0.89, 0.92, 0.95
Proportion hospitalized	Proportion of laboratory-confirmed *S.* Typhimurium illnesses reported to FoodNet that resulted in hospitalization by FoodNet site (n = 10) and year (2017–2019).	Empirical	Minimum, lower quartile, median, upper quartile, maximum values: 0.07, 0.22, 0.29, 0.35, 0.44 Refer to Appendix 2 Table 19 below for a full list of distribution values used.
Proportion who died	Proportion of laboratory-confirmed *S.* Typhimurium illnesses reported to FoodNet that resulted in death by FoodNet site (n = 10) and year (2017–2019).	Empirical	Minimum, lower quartile, median, upper quartile, maximum values: 0, 0, 0, 0.013, 0.042 Refer to Appendix 2 Table 20 below for a full list of distribution values used.
Specimen submission, hospitalizations and deaths	Proportion of hospitalized patients with nonspecific gastroenteritis diagnosis codes who submitted a stool sample for bacterial culture from two published studies (refer to Appendix 1) (*5*,*6*). Uncertainty with this proportion was based on a 50% relative increase/decrease on an odds scale.	PERT	Low, modal, high values: 0.61, 0.70, 0.78
Proportion travel-related	Proportion of laboratory-confirmed *S.* Typhimurium illnesses with reported travel outside the United States within 7 d of illness onset (2017–2019). Uncertainty with this proportion was based on a 50% relative increase/decrease on an odds scale.	PERT	Low, modal, high values: 0.05, 0.07, 0.1
Proportion foodborne	Proportion of domestically acquired *S.* Typhimurium illnesses transmitted through food based on a structured expert judgment study (*7*).	Empirical	Minimum, lower quartile, median, upper quartile, maximum values: 0, 0.48, 0.65, 0.76, 0.95

*NA, not applicable; PERT, program evaluation and review technique.

Appendix 2 Table 13. Nontyphoidal *Salmonella*, other serotypes*

Model input	Data source(s)	Distribution	Distribution values
Reported illnesses	Number of laboratory-confirmed *S.* Other illnesses reported to the Foodborne Diseases Active Surveillance Network (FoodNet) by FoodNet site (n = 10) and year (2017–2019) (*1*). *Salmonella* serotypes with serotype not identified were randomly assigned to one of the six serotype groups with weights equal to the proportions of isolates with known serotype (refer to Appendix 1).	Empirical	Minimum, lower quartile, median, upper quartile, maximum values: 149, 204, 278, 397, 1252 Refer to Appendix 2 Table 17 below for a full list of distribution values.
Population adjustment by year	Population ratios applied to the number of laboratory-confirmed *S.* Other illnesses in each year and FoodNet site combination based on average 2017–2019 U.S. Census population estimates (*2*).	Degenerate	Minimum, lower quartile, median, upper quartile, maximum values: 30.7, 54.1, 77.1, 91.4, 156.1 Refer to Appendix 2 Table 18 below for a full list of distribution values used.
Underreporting	No underreporting multiplier. We assumed that all laboratory-confirmed illnesses were enumerated by FoodNet active surveillance.	NA	NA
Underdiagnosis			
Medical care seeking and specimen submission	Each laboratory-confirmed nontyphoidal *Salmonella* illness in FoodNet surveillance was adjusted for underdiagnosis due to medical care seeking and stool sample submission based on the characteristics of people with acute diarrheal illness who reported seeking medical care and submitting a stool sample in the 2018–2019 FoodNet Population Survey (*3*). Characteristics included age group (<5, 5–64, 65+ years), sex, race and ethnicity (Hispanic; non-Hispanic Black; non-Hispanic white; non-Hispanic other race), and the presence fever and bloody diarrhea. Acute diarrheal illness was defined as diarrhea (≥3 loose stools in 24 h) lasting >1 d or resulting in restricted daily activities, excluding people who said that their illness was due to a long-lasting or chronic illness or condition, such as colitis or irritable bowel syndrome.	Posterior	Refer to Appendix 1
Laboratory testing	We accounted for the percentage of laboratories that routinely tested (on- or off-site) for n*ontyphoidal Salmonella* using data from the FoodNet Laboratory Survey (*4*).	Empirical	Minimum, lower quartile, median, upper quartile, maximum values:0.98, 1, 1, 1, 1
Test sensitivity	Separate adjustments were made for n*ontyphoidal Salmonella* confirmed using culture and culture-independent diagnostic tests (CIDTs). We assumed that CIDTs were 100% sensitive and estimated the sensitivity of culture using reflex culture data from FoodNet. That is, sensitivity of culture was estimated as the proportion of positive reflex cultures among all reflex cultures in FoodNet, and assuming this to be a lower bound, this value was used as the lower parameter of the PERT distribution (refer to Appendix 1).	PERT	Low, modal, high values (culture only): 0.85, 0.89, 0.92
Proportion hospitalized	Proportion of laboratory-confirmed *S.* Other illnesses reported to FoodNet that resulted in hospitalization by FoodNet site (n = 10) and year (2017–2019).	Empirical	Minimum, lower quartile, median, upper quartile, maximum values: 0.15, 0.24, 0.27, 0.30, 0.34 Refer to Appendix 2 Table 19 below for a full list of distribution values used.
Proportion who died	Proportion of laboratory-confirmed *S.* Other illnesses reported to FoodNet that resulted in death by FoodNet site (n = 10) and year (2017–2019).	Empirical	Minimum, lower quartile, median, upper quartile, maximum values: 0, 0.001, 0.004, 0.007, 0.018 Refer to Appendix 2 Table 20 below for a full list of distribution values used.
Specimen submission, hospitalizations and deaths	Proportion of hospitalized patients with nonspecific gastroenteritis diagnosis codes who submitted a stool sample for bacterial culture from two published studies (refer to Appendix 1)	PERT	Low, modal, high values: 0.61, 0.70, 0.78

Model input	Data source(s)	Distribution	Distribution values
	(*5*,*6*). Uncertainty with this proportion was based on a 50% relative increase/decrease on an odds scale.		
Proportion travel-related	Proportion of laboratory-confirmed *S.* Other illnesses with reported travel outside the United States within 7 d of illness onset (2017–2019). Uncertainty with this proportion was based on a 50% relative increase/decrease on an odds scale.	PERT	Low, modal, high values: 0.08, 0.11, 0.16
Proportion foodborne	Proportion of domestically acquired *S.* Other illnesses transmitted through food based on a structured expert judgment study (*7*).	Empirical	Minimum, lower quartile, median, upper quartile, maximum values: 0, 0.38, 0.54, 0.72, 0.99

*NA, not applicable; PERT, program evaluation and review technique.

Appendix 2 Table 14. Shiga toxin-producing *Escherichia coli* (STEC) O157*

Model input	Data source(s)	Distribution	Distribution values
Reported illnesses	Number of laboratory-confirmed STEC O157 illnesses reported to the Foodborne Diseases Active Surveillance Network (FoodNet) by FoodNet site (n = 10) and year (2017–2019) (*1*). STEC isolates with serogroup missing were imputed to O157 or non-O157 with a supervised random forest model using patients' demographics, symptoms, and severity of illness, year of illness, international travel history, and outbreak association (Appendix 1).	Empirical	Minimum, lower quartile, median, upper quartile, maximum values: 18, 33, 56, 86, 208 Refer to Appendix 2 Table 17 below for a full list of distribution values.
Population adjustment by year	Population ratios applied to the number of laboratory-confirmed STEC illnesses in each year and FoodNet site combination based on average 2017–2019 U.S. Census population estimates (*2*).	Degenerate	Minimum, lower quartile, median, upper quartile, maximum values: 30.7, 54.1, 77.1, 91.4, 156.1 Refer to Appendix 2 Table 18 below for a full list of distribution values used.
Underreporting	No underreporting multiplier. We assumed that all laboratory-confirmed illnesses were enumerated by FoodNet active surveillance.	NA	NA
Underdiagnosis			
Medical care seeking and specimen submission	Each laboratory confirmed STEC O157 illness in FoodNet surveillance was adjusted for underdiagnosis due to medical care seeking and stool sample submission based on the characteristics of people with acute diarrheal illness who reported seeking medical care and submitting a stool sample in the 2018–2019 FoodNet Population Survey (*3*). Characteristics included age group (<5, 5–64, 65+ years), sex, race and ethnicity (Hispanic; non-Hispanic Black; non-Hispanic white; non-Hispanic other race), and the presence fever and bloody diarrhea. Acute diarrheal illness was defined as diarrhea (≥3 loose stools in 24 h) lasting >1 d or resulting in restricted daily activities, excluding people who said that their illness was due to a long-lasting or chronic illness or condition, such as colitis or irritable bowel syndrome.	Posterior	Refer to Appendix 1
Laboratory testing	We accounted for the percentage of laboratories that routinely tested (on- or off-site) for STEC O157 using data from the FoodNet Laboratory Survey (*4*).	Empirical	Minimum, lower quartile, median, upper quartile, maximum values:0.90, 0.96, 0.99, 1, 1
Test sensitivity	Separate adjustments were made for STEC O157 illnesses confirmed using culture and culture-independent diagnostic tests (CIDTs). We assumed that CIDTs were 100% sensitive and estimated the sensitivity of culture using reflex culture data from FoodNet. That is, sensitivity of culture was estimated as the proportion of positive reflex cultures among all reflex cultures in FoodNet, and assuming this to be a lower bound, this value was used as the lower parameter of the PERT distribution (Appendix 1).	Degenerate	Value: 1
Proportion hospitalized	Proportion of laboratory confirmed STEC O157 illnesses reported to FoodNet that resulted in hospitalization by FoodNet site (n = 10) and year (2017–2019).	Empirical	Minimum, lower quartile, median, upper quartile, maximum values:

Model input	Data source(s)	Distribution	Distribution values
			0.24, 0.34, 0.42, 0.53, 0.61 Refer to Appendix 2 Table 19 below for a full list of distribution values used.
Proportion who died	Proportion of laboratory confirmed STEC O157 illnesses reported to FoodNet that resulted in death by FoodNet site (n = 10) and year (2017–2019).	Empirical	Minimum, lower quartile, median, upper quartile, maximum values: 0, 0, 0.002, 0.014, 0.049 Refer to Appendix 2 Table 20 below for a full list of distribution values used.
Specimen submission, hospitalizations and deaths	Proportion of hospitalized patients with nonspecific gastroenteritis diagnosis codes who submitted a stool sample for bacterial culture from two published studies (refer to Appendix 1) (*5*,*6*). Uncertainty with this proportion was based on a 50% relative increase/decrease on an odds scale.	PERT	Low, modal, high values: 0.61, 0.70, 0.78
Proportion travel-related	Proportion of laboratory confirmed STEC illnesses with reported travel outside the United States within 7 d of illness onset (2017–2019). Uncertainty with this proportion was based on a 50% relative increase/decrease on an odds scale.	PERT	Low, modal, high values: 0.06, 0.09, 0.13
Proportion foodborne	Proportion (mean and 95% uncertainty interval) of domestically acquired illnesses transmitted through food based on a structured expert judgment study (*7*).	Empirical	Minimum, lower quartile, median, upper quartile, maximum values: 0.30, 0.54, 0.65, 0.73, 0.90

*NA, not applicable; PERT, program evaluation and review technique.

Appendix 2 Table 15. Shiga toxin-producing *Escherichia coli* (STEC) non-O157 strains*

Model input	Data source(s)	Distribution	Distribution values
Reported illnesses	Number of laboratory-confirmed non-O157 STEC illnesses reported to the Foodborne Diseases Active Surveillance Network (FoodNet) by FoodNet site (n = 10) and year (2017–2019) (*1*).	Empirical	Minimum, lower quartile, median, upper quartile, maximum values: 88, 131, 204, 271, 499 Refer to Appendix 2 Table 17 below for a full list of distribution values.
Population adjustment by year	Population ratios applied to the number of laboratory-confirmed non-O157 STEC illnesses in each year and FoodNet site combination based on average 2017–2019 U.S. Census population estimates (*2*).	Degenerate	Minimum, lower quartile, median, upper quartile, maximum values: 30.7, 54.1, 77.1, 91.4, 156.1 Refer to Appendix 2 Table 18 below for a full list of distribution values used.
Underreporting	No underreporting multiplier. We assumed that all laboratory-confirmed illnesses were enumerated by FoodNet active surveillance.	NA	NA
Underdiagnosis			
Medical care seeking and specimen submission	Each laboratory-confirmed non-O157 STEC illness in FoodNet surveillance was adjusted for underdiagnosis due to medical care seeking and stool sample submission based on the characteristics of people with acute diarrheal illness who reported seeking medical care and submitting a stool sample in the 2018–2019 FoodNet Population Survey (*3*). Characteristics included age group (<5, 5–64, 65+ years), sex, race and ethnicity (Hispanic; non-Hispanic Black; non-Hispanic white; non-Hispanic other race), and the presence fever and bloody diarrhea. Acute diarrheal illness was defined as diarrhea (≥3 loose stools in 24 h) lasting >1 d or resulting in restricted daily activities, excluding people who said that their illness was due to a long-lasting or chronic illness or condition, such as colitis or irritable bowel syndrome.	Posterior	Refer to Appendix 1

Model input	Data source(s)	Distribution	Distribution values
Laboratory testing	We accounted for the percentage of laboratories that routinely tested (on- or off-site) for non-O157 STEC using data from the FoodNet Laboratory Survey (*4*).	Empirical	Minimum, lower quartile, median, upper quartile, maximum values: 0.90, 0.96, 0.99, 1, 1
Test sensitivity	Separate adjustments were made for non-O157 STEC illnesses confirmed using culture and culture-independent diagnostic tests (CIDTs). We assumed that CIDTs were 100% sensitive and estimated the sensitivity of culture using reflex culture data from FoodNet. That is, sensitivity of culture was estimated as the proportion of positive reflex cultures among all reflex cultures in FoodNet, and assuming this to be a lower bound, this value was used as the lower parameter of the PERT distribution (Appendix 1).	Degenerate	Value: 1
Proportion hospitalized	Proportion of laboratory-confirmed non-O157 STEC illnesses reported to FoodNet that resulted in hospitalization by FoodNet site (n = 10) and year (2017–2019).	Empirical	Minimum, lower quartile, median, upper quartile, maximum values: 0.06, 0.12, 0.14, 0.19, 0.30 Refer to Appendix 2 Table 19 below for a full list of distribution values used.
Proportion who died	Proportion of laboratory-confirmed non-O157 STEC illnesses reported to FoodNet that resulted in death by FoodNet site (n = 10) and year (2017–2019).	Empirical	Minimum, lower quartile, median, upper quartile, maximum values: 0, 0, 0.002, 0.005, 0.013 Refer to Appendix 2 Table 20 below for a full list of distribution values used.
Specimen submission, hospitalizations and deaths	Proportion of hospitalized patients with nonspecific gastroenteritis diagnosis codes who submitted a stool sample for bacterial culture from two published studies (refer to Appendix 1) (*5*,*6*). Uncertainty with this proportion was based on a 50% relative increase/decrease on an odds scale.	PERT	Low, modal, high values: 0.61, 0.70, 0.78
Proportion travel-related	Proportion of laboratory-confirmed *Escherichia coli* non-O157 illnesses with reported travel outside the United States within 7 d of illness onset (2017–2019). Uncertainty with this proportion was based on a 50% relative increase/decrease on an odds scale.	PERT	Low, modal, high values: 0.17, 0.23, 0.31
Proportion foodborne	Proportion (mean and 95% uncertainty interval) of domestically acquired illnesses transmitted through food based on a structured expert judgment study (*7*).	Empirical	Minimum, lower quartile, median, upper quartile, maximum values: 0.20, 0.38, 0.55, 0.69, 0.95

*NA, not applicable; PERT, program evaluation and review technique.

Appendix 2 Table 16. *Toxoplasma gondii**

Model input	Data source(s)	Distribution	Parameters
Hospitalizations	Rate of hospitalizations per 100,000 from the 2016–2019 National Inpatient Sample (NIS) using ICD-9-CM code B58 (Toxoplasmosis).	Empirical	By year 2016–2019: 0.67, 0.63, 0.61, 0.63
Deaths	Rate of toxoplasmosis inpatient deaths per 100,000 from the 2016–2019 NIS using ICD-9-CM code B58 (Toxoplasmosis).	Empirical	By year 2016–2019: 0.03, 0.03, 0.03, 0.04
Population adjustment by year	Estimates applied to the Census population 2017–2019 (*2*).		Adjustment by year (2016–2019): 1.01, 1.01, 1, 1
Underdiagnosis, hospitalizations and deaths	Underdiagnosis multiplier for *Salmonella*, non-typhoidal hospitalizations and deaths applied.	NA	NA
Proportion travel-related	Assumed to be very low.	PERT	Low, modal, high values: 0, 0, 0.2

Model input	Data source(s)	Distribution	Parameters
Proportion foodborne	Proportion (mean and 95% uncertainty interval) of domestically acquired *Toxoplasma gondii* illnesses transmitted through food based on a structured expert judgment study (*7*).	Empirical	Minimum, lower quartile, median, upper quartile, maximum values: 0.01, 0.14, 0.24, 0.48, 0.88

*NA, not applicable; PERT, program evaluation and review technique.

Appendix 2 Table 17. Number of illnesses reported to Foodborne Diseases Active Surveillance Network (FoodNet) according to pathogen, year, and US state*

	FoodNet sites									
Pathogen	CA	CO	CT	GA	MD	MN	NM	NY	OR	TN
Campylobacter spp.										
2017	1,279	562	659	1,179	864	1,554	760	679	1,072	908
2018	1,306	655	743	1,417	950	1,569	611	690	969	922
2019	1,237	647	724	1,395	886	1,489	686	794	1,018	923
STEC O157										
2017	81	42	33	80	47	190	18	33	88	123
2018	62	27	33	113	50	208	20	44	90	108
2019	76	47	31	78	42	194	24	20	82	76
STEC, non-O157										
2017	210	180	95	287	153	343	88	95	203	198
2018	212	199	106	351	204	399	102	124	207	256
2019	276	238	103	401	192	499	95	156	221	293
Salmonella serotype Enteritidis										
2017	115	66	121	396	215	249	46	109	88	189
2018	118	76	109	428	207	285	50	114	102	183
2019	107	86	145	383	240	216	53	130	92	190
Salmonella serotype I 4,[5],12:i:-										
2017	44	39	25	100	74	100	20	41	33	68
2018	35	29	30	125	47	90	25	45	42	55
2019	45	18	21	95	52	77	16	32	27	67
Salmonella serotype Javiana										
2017	17	12	24	317	109	19	28	13	25	79
2018	24	14	15	484	114	24	33	17	19	119
2019	17	12	19	390	91	30	32	21	14	107
Salmonella serotype Newport										
2017	33	29	26	366	79	56	53	47	24	101
2018	68	59	34	423	110	41	91	33	46	127
2019	41	32	37	366	113	53	49	39	34	114
Salmonella serotype Typhimurium										
2017	52	66	62	174	89	98	52	46	67	123
2018	68	46	71	212	102	112	42	63	75	180
2019	67	41	60	198	103	75	35	48	62	133
Other nontyphoidal *Salmonella* spp.†										
2017	329	168	203	982	311	417	149	199	207	398
2018	293	168	240	1,252	352	492	164	262	238	448
2019	359	191	232	1,239	395	367	173	219	213	489

*Foodborne Diseases Active Surveillance Network (https://www.cdc.gov/foodnet). Data from California, Colorado, and New York were from selected counties. CA, California; CO, Colorado; CT, Connecticut; GA, Georgia; MD, Maryland; MN, Minnesota; NM, New Mexico; NY, New York; OR, Oregon; STEC, Shiga toxin–producing *Escherichia coli*; TN, Tennessee.
†*Salmonella* serotype Paratyphi was excluded from all analyses.

Appendix 2 Table 18. Ratios of average 2017–2019 US census population to populations in the Foodborne Diseases Active Surveillance Network (FoodNet) database according to year and US state*

	FoodNet sites									
Year	CA	CO	CT	GA	MD	MN	NM	NY	OR	TN
2017	88.72	103.49	91.39	31.37	54.21	58.67	156.13	76.27	78.79	48.66
2018	88.38	102.15	91.41	31.06	54.08	58.26	156.07	76.35	78.11	48.21
2019	88.31	101.18	91.63	30.75	53.97	57.94	155.63	76.60	77.50	47.84

*Foodborne Diseases Active Surveillance Network (https://www.cdc.gov/foodnet). Data from California, Colorado, and New York were from selected counties. Ratios were written as a single number for simplicity. For example, 88.72 denotes a ratio of 88.72:1. CA, California; CO, Colorado; CT, Connecticut; GA, Georgia; MD, Maryland; MN, Minnesota; NM, New Mexico; NY, New York; OR, Oregon; TN, Tennessee. Ratios were written as a single number for simplicity. For example, 88.72 denotes a ratio of 88.72:1.

Appendix 2 Table 19. Proportions of laboratory-confirmed illnesses resulting in hospitalization from the Foodborne Diseases Active Surveillance Network (FoodNet) according to pathogen, year, and US state*

	FoodNet sites*									
Pathogen	CA	CO	CT	GA	MD	MN	NM	NY	OR	TN
Campylobacter spp.										
2017	0.0987	0.1921	0.1905	0.3440	0.2473	0.1718	0.2582	0.2407	0.1290	0.3416
2018	0.0961	0.1296	0.2081	0.3231	0.2241	0.1504	0.2385	0.2143	0.1277	0.3203
2019	0.1221	0.1869	0.2067	0.3423	0.2305	0.1592	0.2390	0.2406	0.1398	0.3146
STEC O157										
2017	0.3266	0.5139	0.5298	0.5860	0.4705	0.2883	0.4394	0.2448	0.3438	0.4531
2018	0.2533	0.4892	0.4256	0.5530	0.3636	0.2556	0.5364	0.4162	0.4016	0.4243
2019	0.3245	0.3744	0.5624	0.5794	0.3395	0.3440	0.6025	0.5955	0.2986	0.6126
STEC, non-O157										
2017	0.0826	0.1366	0.1758	0.3048	0.1660	0.1345	0.1164	0.1994	0.1173	0.1627
2018	0.1219	0.1142	0.1425	0.2481	0.1460	0.1676	0.1397	0.1983	0.1439	0.2358
2019	0.0616	0.1301	0.1827	0.2288	0.1482	0.1368	0.1146	0.2053	0.1194	0.2561
Salmonella serotype Enteritidis										
2017	0.1481	0.1875	0.2397	0.3437	0.3380	0.2088	0.4130	0.2661	0.2159	0.2663
2018	0.1930	0.2400	0.3578	0.3155	0.3592	0.2281	0.2600	0.2807	0.2400	0.2711
2019	0.1635	0.2738	0.2690	0.3699	0.3870	0.2454	0.3019	0.2615	0.1848	0.3812
Salmonella serotype I 4,[5],12:i:-										
2017	0.1463	0.2564	0.3600	0.3814	0.3014	0.2200	0.3500	0.4634	0.2121	0.4500
2018	0.1429	0.1379	0.3667	0.3478	0.3478	0.2222	0.2400	0.4000	0.0952	0.4615
2019	0.1628	0.1765	0.3333	0.2778	0.3673	0.2597	0.2500	0.3125	0.1111	0.4918
Salmonella serotype Javiana										
2017	0.0000	0.1667	0.2917	0.3097	0.3107	0.1579	0.3214	0.1667	0.2083	0.2833
2018	0.1739	0.2143	0.2000	0.3211	0.2364	0.1250	0.1818	0.2353	0.1053	0.3365
2019	0.0000	0.1667	0.4211	0.3085	0.3111	0.3000	0.2812	0.2857	0.2143	0.3789
Salmonella serotype Newport										
2017	0.1562	0.1724	0.4615	0.3249	0.2895	0.2679	0.3396	0.3404	0.2917	0.2651
2018	0.1094	0.2759	0.3333	0.2727	0.1981	0.1951	0.4176	0.3939	0.2174	0.2700
2019	0.1026	0.2903	0.3514	0.3371	0.2736	0.1321	0.2653	0.2368	0.1818	0.3269
Salmonella serotype Typhimurium										
2017	0.1739	0.2769	0.3710	0.2798	0.3412	0.1735	0.3846	0.3043	0.2090	0.3486
2018	0.0735	0.1111	0.2958	0.2526	0.4343	0.2946	0.2927	0.2698	0.2533	0.3861
2019	0.1667	0.1220	0.2667	0.2880	0.3505	0.2933	0.4286	0.3958	0.1935	0.4390
Other nontyphoidal *Salmonella* spp.										

Pathogen	FoodNet sites*									
	CA	CO	CT	GA	MD	MN	NM	NY	OR	TN
2017	0.1516	0.2683	0.3448	0.2737	0.2614	0.2494	0.2381	0.2714	0.2367	0.2720
2018	0.1972	0.2515	0.3389	0.2921	0.2515	0.2398	0.3049	0.2748	0.2110	0.3116
2019	0.1749	0.2500	0.3190	0.3241	0.3113	0.2071	0.3000	0.2648	0.2238	0.3185

*Foodborne Diseases Active Surveillance Network (https://www.cdc.gov/foodnet). Data from California, Colorado, and New York were from selected counties. CA, California; CO, Colorado; CT, Connecticut; GA, Georgia; MD, Maryland; MN, Minnesota; NM, New Mexico; NY, New York; OR, Oregon; STEC, Shiga toxin–producing *Escherichia coli*; TN, Tennessee.

Appendix 2 Table 20. Proportions of laboratory-confirmed illnesses resulting in death from the Foodborne Diseases Active Surveillance Network (FoodNet) according to pathogen, year, and US state*

Pathogen	FoodNet site*									
	CA	CO	CT	GA	MD	MN	NM	NY	OR	TN
Campylobacter spp.										
2017	0.0010	0.0036	0	0.0066	0.0083	0.0039	0.0066	0.0029	0.0009	0.0047
2018	0.0030	0.0031	0.0013	0.0074	0.0032	0.0019	0.0033	0.0029	0.0021	0.0093
2019	0.0022	0	0.0028	0.0102	0.0023	0.0007	0.0029	0.0025	0.0010	0.0034
STEC O157										
2017	0	0.0018	0.0302	0.0046	0	0.0029	0.0489	0	0	0.0139
2018	0	0.0393	0.0061	0.0016	0	0.0123	0.0147	0.0225	0	0.0144
2019	0	0.0035	0	0.0325	0	0	0.0425	0.0134	0.0012	0
STEC, non-0157										
2017	0	0.0052	0.0000	0.0022	0	0.0042	0.0129	0	0	0.0018
2018	0	0.0047	0.0075	0.0052	0	0.0036	0.0069	0.0081	0	0.0025
2019	0	0.0035	0	0.0061	0	0	0	0.0047	0.0086	0
Salmonella serotype Enteritidis										
2017	0	0.0312	0	0.0077	0	0.0040	0	0	0.0114	0.0055
2018	0.0094	0	0	0.0095	0.0097	0.0070	0	0	0.0098	0
2019	0.0000	0.0235	0	0.0161	0	0	0	0.0154	0	0.0160
Salmonella serotype I 4,[5],12:i:-										
2017	0	0.0769	0	0.0101	0	0	0	0	0	0.0169
2018	0	0	0	0	0.0638	0.0111	0	0	0	0
2019	0	0	0	0	0.0208	0	0	0	0	0
Salmonella serotype Javiana										
2017	0	0	0	0.0032	0	0	0	0	0	0
2018	0	0	0	0.0063	0	0	0	0	0	0
2019	0	0	0	0	0	0.0333	0	0	0	0.0100
Salmonella serotype Newport										
2017	0	0	0	0	0	0.0179	0	0	0	0
2018	0	0	0	0.0048	0	0	0.0110	0	0	0
2019	0	0.0312	0	0.0028	0.0091	0	0	0	0	0
Salmonella serotype Typhimurium										
2017	0	0	0	0	0	0	0	0	0	0.0171
2018	0.0156	0	0	0.0097	0	0.0179	0	0	0.0133	0.0059
2019	0.0217	0	0.0333	0.0052	0.0101	0.0133	0	0.0417	0	0.0079
Other nontyphoidal *Salmonella* spp.										
2017	0	0	0.0049	0.0041	0.0033	0.0048	0.0068	0.0151	0.0048	0.0054
2018	0	0	0.0042	0.0057	0	0	0.0122	0	0.0042	0.0024
2019	0.0093	0	0.0129	0.0073	0.0078	0.0027	0.0175	0.0046	0.0141	0.0022

Pathogen	FoodNet site*									
	CA	CO	CT	GA	MD	MN	NM	NY	OR	TN

*Foodborne Diseases Active Surveillance Network (https://www.cdc.gov/foodnet). Data from California, Colorado, and New York were from selected counties. CA, California; CO, Colorado; CT, Connecticut; GA, Georgia; MD, Maryland; MN, Minnesota; NM, New Mexico; NY, New York; OR, Oregon; STEC, Shiga toxin–producing *Escherichia coli*; TN, Tennessee.

References

1. Centers for Disease Control and Prevention. Foodborne Diseases Active Surveillance Network. About FoodNet. 2024 [cited 2024 Apr 3]. https://www.cdc.gov/foodnet/surveillance.html

2. United States Census Bureau. Population estimates. 2024 [cited 2023 Oct 5]. https://www2.census.gov/programs-surveys/popest/datasets

3. Devine CJ, Molinari NA, Shah HJ, Blackstock AJ, Geissler A, Marder EP, et al. The 2018–2019 FoodNet Population Survey: a tool to estimate risks and behaviors associated with enteric infections. Am J Epidemiol. 2025;194:5–11. PubMed https://doi.org/10.1093/aje/kwae127

4. Ray LC, Griffin PM, Wymore K, Wilson E, Hurd S, LaClair B, et al. Changing diagnostic testing practices for foodborne pathogens, Foodborne Diseases Active Surveillance Network, 2012–2019. Open Forum Infect Dis. 2022;9:ofac344. PubMed https://doi.org/10.1093/ofid/ofac344

5. Scallan Walter EJ, McLean HQ, Griffin PM. Hospital discharge data underascertain enteric bacterial infections among children. Foodborne Pathog Dis. 2020;17:530–2. PubMed https://doi.org/10.1089/fpd.2019.2773

6. Scallan E, Griffin PM, McLean HQ, Mahon BE. Hospitalisations due to bacterial gastroenteritis: a comparison of surveillance and hospital discharge data. Epidemiol Infect. 2018;146:954–60. PubMed https://doi.org/10.1017/S0950268818000882

7. Beshearse E, Bruce BB, Nane GF, Cooke RM, Aspinall W, Hald T, et al. Attribution of illnesses transmitted by food and water to comprehensive transmission pathways using structured expert judgment, United States. Emerg Infect Dis. 2021;27:182–95. PubMed https://doi.org/10.3201/eid2701.200316

8. Centers for Disease Control and Prevention. Foodborne disease outbreak surveillance system. 2024 [cited 2024 Apr 3]. https://www.cdc.gov/nors/about/fdoss.html

9. Centers for Disease Control and Prevention. About the *Listeria* Initiative. 2024 [cited 2024 Apr 3]. https://www.cdc.gov/listeria/php/surveillance/listeria-initiative.html

10. Burke RM, Mattison CP, Marsh Z, Shioda K, Donald J, Salas SB, et al. Norovirus and other viral causes of medically attended acute gastroenteritis across the age spectrum: results from the medically attended acute gastroenteritis study in the United States. Clin Infect Dis. 2021;73:e913–20. PubMed https://doi.org/10.1093/cid/ciab033

11. Burke RM, Mattison CP, Pindyck T, Dahl RM, Rudd J, Bi D, et al. Burden of norovirus in the United States, as estimated based on administrative data: updates for medically attended illness and mortality, 2001–2015. Clin Infect Dis. 2021;73:e1–8. PubMed https://doi.org/10.1093/cid/ciaa438
