# Supplementary material for: Foodborne Illness Acquired in the United States—Major Pathogens, 2019
Source: Emerg Infect Dis. 2025 Apr;31(4):669–77. doi: 10.3201/eid3104.240913 (PMC11950263; doi:10.3201/eid3104.240913)
Supplement: Appendix 3 — Additional information for means and credible intervals used for foodborne illness acquired in the United States—major pathogens, 2019. [file 24-0913-Techapp-s3.pdf]

Article DOI. https://doi.org/10.3201/eid3104.240913
EID cannot ensure accessibility for supplementary materials supplied by authors. Readers who have difficulty accessing supplementary content should contact the authors for assistance.

Foodborne Illness Acquired in the United States—Major Pathogens, 2019

Appendix 3

Tables with Unrounded Totals, Means, and Credible Intervals

Appendix 3 Table 1. Estimated annual number of illnesses caused by *Campylobacter* spp., *Clostridium perfringens*, invasive *Listeria monocytogenes*, norovirus, *Salmonella* spp. (nontyphoidal), and Shiga toxin-producing *Escherichia coli*, United States, circa 2019*

Pathogen	Laboratory confirmed†	Multipliers: Underreporting	Multipliers: Underdiagnosis	Total no., mean (90% CrI)	% from international travel	% Foodborne	No. domestically acquired foodborne illnesses, mean (90% CrI)
Campylobacter spp.	72,218‡	1	52.8	4,019,869 (1,785,702–7,498,912)	19	57	1,872,423 (696,479–3,755,174)
Clostridium perfringens	811§	28.9	38.1	891,671 (19,381–3,105,774)	<1	100	888,530 (19,305–3,091,328)
Listeria moncytogenes, invasive¶				1,291 (1,112–1,506)	NA	NA	1,250 (1,076–1,458)
NPA	656#	1	1.7	1,087 (941–1,260)	3	100	1,052 (911–1,220)
PA, mothers	66#	1	1.7	110 (79–139)	3	100	106 (76–134)
PA, infants	57#	1	1.7	95 (76–126)	3	99	92 (74–122)
Norovirus	2,915,705**	1	10.1	29,361,639 (21,187,581–38,710,145)	1	19	5,539,708 (2,225,981–10,264,359)
Nontyphoidal *Salmonella* spp. serotypes¶				2,413,847 (1,832,091–3,126,335)	NA	NA	1,280,960 (865,621–1,761,268)
Enteritidis	11,240‡	1	38.8	479,055 (302,325–720,869)	22	80	297,182 (181,397–452,599)
I 4,[5],12:i-	3,721‡	1	38.5	159,344 (89,236–266,424)	10	66	93,898 (46,515–163,478)
Javiana	4,324‡	1	39.2	172,465 (46,161–387,390)	5	56	91,805 (21,424–214,934)
Newport	5,906‡	1	38.4	260,644 (102,989–679,055)	7	74	179,069 (66,229–462,706)
Typhimurium	6,287‡	1	39.0	248,881 (148,725–384,778)	7	59	135,136 (63,026–226,812)
Other	24,504‡	1	39.6	1,093,459 (703,336–1,611,006)	11	50	483,870 (171,465–853,096)
STEC#				868,744 (436,429–1,463,209)	NA	NA	357,200 (158,682–648,489)
O157	5,069‡	1	38.9	159,894 (66,131–362,008)	9	60	86,228 (31,355–201,197)
Non-O157	15,648‡	1	42.7	708,850 (323,359–1,234,805)	24	50	270,972 (97,166–545,855)
All pathogens¶				37,557,061 (28,466,598–47,530,146)	NA	NA	9,940,072 (5,863,417–15,422,269)

*Actual numbers. CrI, credible interval; NA, not applicable; NPA, non-pregnancy associated; PA, pregnancy associated; STEC, Shiga toxin–producing *Escherichia coli*; Int'l travel, international travel.
†Estimates according to the average US population during 2017–2019.
‡Active surveillance data from Foodborne Diseases Active Surveillance Network (2017–2019), adjusted for geographic coverage.
§Passive surveillance data on outbreak-associated illnesses from the Foodborne Disease Outbreak Surveillance System (2010–2019).
¶Total number of illnesses and the number of domestically acquired foodborne illness were the sum of the estimates for corresponding subgroups.
#Passive surveillance data from the *Listeria* Initiative (2017–2019).
**Active surveillance from Kaiser Permanente Northwest (2014–2016) and administrative data from IBM® MarketScan® Commercial and Medicare Supplemental Databases (2001–2015) (*1,2*) applied to the U.S. population during 2017–2019.

Appendix 3 Table 2. Estimated annual number of hospitalizations and deaths caused by *Campylobacter* spp., *Clostridium perfringens*, invasive *Listeria monocytogenes*, norovirus, *Salmonella* (non-typhoidal), Shiga toxin-producing *Escherichia coli*, and *Toxoplasma gondii*, United States, circa 2019*

	Hospitalizations				Deaths			
Pathogen	%	Underdiagnosis multiplier	Total no., mean (90% CrI)†	No. domestically acquired foodborne illnesses, mean (90% CrI)†	%	Underdiagnosis multiplier	Total no., mean (90% CrI)†	No. domestically acquired foodborne illnesses, mean (90% CrI)†
Campylobacter spp.	22.1‡	1.7	27,853 (12,165–51,110)	12,983 (4,735–25,492)	0.3‡	1.7	420 (0–1,178)	197 (0–585)
Clostridium perfringens	<1§	1.5	339 (0–1,935)	338 (0–1,922)	0.1§	1.5	41 (0–190)	40 (0–189)
Listeria moncytogenes, invasive¶			1,103 (953–1,285)	1,068 (922–1,244)	NA	NA	178 (148–210)	172 (143–203)
NPA	87.4#	1.7	950 (822–1,101)	920 (796–1,067)	15.8#	1.7	172 (146–202)	166 (142–195)
PA, mothers	70.4#	1.7	77 (60–94)	74 (58–91)	0#	1.7	0	0
PA, infants	80.6#	1.7	77 (60–106)	74 (58–103)	5.8#	1.7	6 (0–11)	6 (0–10)
Norovirus**	<0.1	–	118,695 (96,876–141,133)	22,372 (9,570–39,911)	<0.001	–	924 (788–1,071)	174 (76–308)
Nontyphoidal *Salmonella* spp. serotypes¶			23,492 (17,132–31,579)	12,545 (8,248–17,733)	NA	NA	446 (15–1,092)	238 (7–602)
Enteritidis	27.7‡	1.5	4,760 (2,546–7,854)	2,966 (1,532–4,983)	0.6‡	1.5	99 (0–383)	63 (0–242)
I 4,[5],12:i-	28.7‡	1.5	1,622 (645–2,903)	957 (339–1,765)	0.7‡	1.5	43 (0–263)	26 (0–158)
Javiana	25.2‡	1.5	1,655 (174–4,821)	882 (79–2,620)	0.1‡	1.5	9 (0–56)	5 (0–30)
Newport	28.0‡	1.5	2,498 (787–5,689)	1,720 (507–3,997)	0.3‡	1.5	24 (0–166)	17 (0–116)
Typhimurium	29.3‡	1.5	2,800 (1,258–4,657)	1,522 (576–2,697)	0.8‡	1.5	73 (0–348)	40 (0–191)
Other	27.1‡	1.5	10,157 (6,196–15,745)	4,498 (1,546–8,181)	0.5‡	1.5	197 (0–633)	88 (0–309)
STEC¶			6,922 (3,423–12,243)	3,146 (1,438–5,871)	NA	NA	141 (0–442)	66 (0–217)
O157	43.1‡	1.5	3,208 (1,113–7,572)	1,732 (574–4,075)	1.0‡	1.5	75 (0–321)	40 (0–180)
Non-O157	16.2‡	1.5	3,714 (1,526–7,653)	1,414 (465–3,018)	0.3‡	1.5	66 (0–230)	25 (0–94)
Toxoplasma gondii††	<0.001	1.5	3,182 (2,792–3,562)	848 (173–1,699)	<0.001	1.5	163 (134–217)	43 (9–90)
All pathogens¶			181,587 (151,570–214,517)	53,299 (35,771–74,560)	NA	NA	2,313 (1,581–3,278	931 (530–1,460)

*Actual numbers. CrI, credible interval; NA, not applicable; NPA, nonpregnancy associated; PA, pregnancy associated; STEC, Shiga toxin–producing *Escherichia coli*.
†Estimates according to the average US population during 2017–2019.
‡Percentage hospitalized and died from the Foodborne Diseases Active Surveillance Network (2017–2019)
§Percentage hospitalized and died from the Foodborne Disease Outbreak Surveillance System (2010–2019).
¶Total number of illnesses and the number of domestically acquired foodborne illness were the sum of the estimates for corresponding subgroups.
#Percentages from the US Centers for Disease Control and Prevention's *Listeria* Initiative (2017–2019). We estimated an additional 38 (90% CrI 28–47) fetal deaths, of which 37 (90% CrI 27–45) were estimated to be from domestically acquired, foodborne illnesses.
**Rates of hospitalizations and deaths from the National Inpatient Sample of the Healthcare Cost and Utilization Project (hospitalizations) and the National Center for Health Statistics (NCHS) multiple-cause-of-mortality dataset (deaths) for norovirus (*2*) applied to the U.S. population during 2017–2019.
††According to the rates of hospitalizations and deaths from the National Inpatient Sample (2016–2019) applied to the US population during 2017–2019.

References

1. Burke RM, Mattison CP, Marsh Z, Shioda K, Donald J, Salas SB, et al. Norovirus and other viral causes of medically attended acute gastroenteritis across the age spectrum: results from the medically attended acute gastroenteritis study in the United States. Clin Infect Dis. 2021;73:e913–20. PubMed https://doi.org/10.1093/cid/ciab033

2. Burke RM, Mattison CP, Pindyck T, Dahl RM, Rudd J, Bi D, et al. Burden of norovirus in the United States, as estimated based on administrative data: updates for medically attended illness and mortality, 2001–2015. Clin Infect Dis. 2021;73:e1–8. PubMed https://doi.org/10.1093/cid/ciaa438
